# Rituals and knowledge of Andean midwives in maternal and neonatal care

**DOI:** 10.3389/fgwh.2026.1857301

**Published:** 2026-07-14

**Authors:** Edgar Gutiérrez-Gómez, Yolanda Justina Soliz-Yaranga, Rubén Darío Alania-Contreras, Daniela Isabel Dayan Ortega-Révolo, Wilfredo Bazán-Ramírez, Aldo Bazán-Ramírez

**Affiliations:** 1 Faculty of Engineering and Management, School of Sustainable Tourism and Hospitality Management, National Autonomous University of Huanta, Ayacucho, Peru; 2 Private Higher Pedagogical School of Education “Cuna de la Libertad Americana”, Ayacucho, Perú; 3 Faculty of Communication Sciences, National University of Central Peru, Junín, Perú; 4 Faculty of Business Sciences, Professional Academic School of Economics, Continental University, Huancayo, Peru; 5 Faculty of Administrative Sciences, National University of San Marcos, Lima, Perú; 6 Professional School of Psychology, José María Arguedas National University, Andahuaylas, Perú

**Keywords:** ancestral knowledge, andean culture, Andean midwives, birth rituals, global maternal health, intercultural health, quechua

## Abstract

**Introduction:**

This study examines the persistence of the knowledge and rituals of Andean midwives within a context of medicalization that has progressively delegitimized ancestral knowledge systems and traditional forms of maternal and neonatal care among rural Quechua-speaking populations in Ayacucho, Peru. The study is framed within the contemporary challenges of global maternal health and intercultural healthcare in Indigenous communities.

**Objective:**

To analyze how the knowledge and ritual practices of Andean midwives shape maternal and neonatal care and how these practices are articulated with biomedicine.

**Method:**

A qualitative ethnographic approach was employed, based on in-depth interviews with 20 participants: 8 traditional birth attendants, including seven women and one man, 7 mothers, and 5 community actors involved in maternal and neonatal care. Data collection also included participant observation and field notes conducted in rural communities of Ayacucho.

**Results:**

The findings reveal a therapeutic system grounded in thermal-body balance, the use of medicinal plants, family accompaniment, ritual practices, and the intergenerational transmission of knowledge. Tensions with the biomedical system were identified, characterized by processes of partial delegitimization, communication barriers, and selective adaptation between traditional and institutional healthcare systems.

**Discussion:**

The knowledge of Andean midwives constitutes a living system of care that ensures comprehensive attention, cultural continuity, and community trust during pregnancy, childbirth, and postpartum care. Their recognition and integration into intercultural health policies are essential for reducing healthcare disparities and strengthening maternal and neonatal care in rural Indigenous societies.

## 1 Introduction

Andean communities in Peru have their own internal organization; some are considered peasant communities with local autonomy in territorial management, since land is communally owned. Political and social organization is carried out by the community members themselves, given that this “cultural awareness translates into greater respect for cultural practices and a greater willingness to preserve and protect the community's cultural heritage, as well as the generation of direct economic income for the local population” (1). This cultural awareness strengthens identity, promotes heritage preservation, and enhances the local economy through the sustainable valorization of traditional community practices. Within their internal organization, they preserve and value ancestral knowledge related to childbirth and newborn care, where “the traditional midwife is a woman who, from her system of knowledge, provides a service to the community for the care of pregnancy, childbirth, and the postpartum period” (2). In this way, the traditional midwife is recognized as a community agent who provides maternal care based on her own system of knowledge, which is socially legitimized.

Older people, especially women, recall that childbirth and newborn care were carried out according to their own customs, and not as indicated by contemporary medicine. The latter tends to progressively displace ancestral knowledge related to motherhood, where “many of these customs that are part of ancestral knowledge have survived thanks to oral tradition and the survival of individuals who have taken responsibility for continuing these traditional forms of knowledge” (3). Oral tradition and key actors are therefore essential for preserving and transmitting this culturally significant knowledge. The fact that these traditions are preserved orally and in Quechua, a language without a formal written system, contributes to the devaluation of these childbirth practices, granting greater legitimacy to modern science. It has been stated that “Western medicine may affect Indigenous populations by failing to recognize their culture and ancestral care practices, as well as by lacking prior communication that could strengthen empathy and trust in the doctor–patient relationship” (4). Western biomedical care may generate cultural tensions in Indigenous populations when traditional practices and knowledge are not considered within healthcare processes. From the participants' perspective, limited intercultural communication and the lack of recognition of traditional practices influence trust in healthcare services.

The incorporation of Western medicine has led to a decrease in childbirths carried out according to ancestral practices by community midwives; currently, in Peru, childbirth care is the responsibility of a health professional. In towns with larger populations, there is usually a health post, and pregnant women are assisted from the earliest stages of pregnancy. Traditionally, however, “preparation for childbirth begins during pregnancy, through traditional knowledge learned among women in the community and supported by the husband” (5). This shows that childbirth constitutes a community-based process, grounded in the female transmission of ancestral knowledge and active spousal co-responsibility. It is important to note that “midwives, since time immemorial and in all peoples of the world, have accompanied other women throughout all stages of their lives. They support childbirth, child-rearing, life-cycle rituals, and when girls experience their menarche” (6), highlighting their universal role in childbirth care, upbringing, and female transitional rituals.

In scientific literature and medical practice, there is no evidence that recognizes the contribution of ancestral medicine to the work of midwives in Andean communities. Their knowledge, preserved in collective memory, has been devalued by Western science, disregarding traces of their practice. Considering this lack of recognition of traditional medicine in childbirth, it becomes necessary that “national and provincial health authorities, as well as public health and medical universities, reassess the ways in which Indigenous medical interventions are valued, especially when applied within their own communities” (7). This emphasizes that health authorities and universities must recognize, reassess, and appropriately value Indigenous community-based medical interventions. The process of interculturality requires understanding the essence of Andean medicine, transmitted orally, since “cultural differences constitute a key social determinant that influences both health behavior and the quality of care received” (8). This highlights that cultural differences are a fundamental social determinant, directly influencing health behaviors and the quality of medical care provided.

This study analyzes the ancestral knowledge of Andean midwives in maternal and neonatal care from an ethnographic perspective. Although various studies have addressed Andean traditional medicine and the role of midwives in rural settings, there is still limited ethnographic evidence on the continuity of their practices, knowledge, and forms of care in rural communities of Ayacucho. This research contributes to the understanding of the ritual practices, knowledge systems, and care practices developed by traditional midwives, highlighting their cultural relevance and their importance within intercultural health processes. In Ayacucho, “in 1997, the international non-governmental organization Health Unlimited began working in isolated communities of Ayacucho, initially establishing links between local communities and the health system through the training of volunteer community health agents” (9). These processes of institutional and biomedical articulation have generated gradual transformations in traditional childbirth practices, partially displacing ancestral knowledge associated with “natural childbirth,” a term used by local rural populations themselves.

## 2 Methodology

The research is based on a qualitative ethnographic approach aimed at understanding the ancestral knowledge and practices of Andean midwives in self-managed communities in the Ayacucho region, specifically in the province of San Miguel, district of Patibamba, a predominantly Quechua-speaking community where Quechua is the main system of communication and is recognized as the second official language of Peru. The study design is descriptive-interpretative, focused on the reconstruction of meanings and current practices related to maternal and neonatal care, understood as an essential component of social and cultural life in Andean communities. The sample consisted of 20 participants (*N* = 20), selected through purposive sampling. Participants included 8 traditional midwives —including one man—, 7 mothers, and 5 community actors involved in maternal and neonatal care. The midwives were between 38 and 82 years old and had between 10 and more than 50 years of experience in childbirth care and ancestral therapeutic practices. The mothers were between 20 and 41 years old, while the community actors ranged from 35 to 74 years old. Coexistence and repeated visits over a six-month period facilitated the development of trust with the rural population. In this process, interlocutors expressed: *kutimuchkanku kaqlla* (they are coming back again) and *chay wachakuikmanta tapukuq* (asking about the old way of childbirth). In the community, childbirth is referred to as *wachakui* or *unqukuy*.

Data collection techniques included in-depth interviews conducted with midwives, adult mothers, and families who practice natural childbirth or who have been attended by midwives; to a lesser extent, men who perform the role of traditional birth attendants were also included. Participant observation was used during the care of pregnant women who visit midwives, particularly in practices such as fetal positioning and the application of traditional medicinal treatments. The information was complemented through informal conversations and field notes focused on local narratives. Data saturation was reached when interviews and observations began to show repetition of discourses, practices, and meanings related to midwives' knowledge, with no emergence of new relevant analytical categories. This point was identified approximately after the 17th interview; however, data collection continued until completed *N*=20 participants in order to confirm the consistency and recurrence of the findings.

For data processing, a thematic-categorical analysis was employed, which allowed the identification of recurrent patterns such as rituality, thermal-body care, the use of medicinal plants, and intercultural tensions with conventional medicine. The process continued through axial coding, establishing relationships between emerging categories and interpretative patterns linked to spirituality, cultural memory, community care, and intercultural health. A thematic interpretation phase was developed, in which categories were integrated into central themes that structured the study's findings. In the ethical domain, informed consent, participant confidentiality, and respect for local knowledge as a cultural expression were ensured. The transcription of interviews and informal dialogues from oral Quechua into Spanish was preserved, in essence, to maintain the integrity and original meaning of the participants' discourses. The researchers, who are Quechua speakers, carried out the initial translation and drafting in Spanish and subsequently in English. Since participants often combine Quechua and Spanish expressions in their speech, some formulations may present certain linguistic limitations inherent to the intercultural translation process. The study employed data triangulation and qualitative techniques in order to strengthen methodological rigor, interpretative reliability, and validity of the findings. The information obtained through in-depth interviews was systematically contrasted with participant observation records and field notes produced during the ethnographic fieldwork.

A marked reluctance was identified among key actors to participate in the research, particularly at the moment of presenting informed consent, which resulted in evasive responses expressed in phrases such as “*manam yachanichu*” (I do not know more details). This reluctance is linked to the perception that their ancestral practice as midwives may be subject to questioning and possible social or institutional sanctions. The potential publication of their names or community is interpreted as a risk, as they could be stigmatized as resistant to change and associated with possible impacts on maternal and neonatal health. In response to this scenario, ethnographic strategies of gradual approach were adopted, where effective data collection was achieved through informal dialogues after extended periods of coexistence in the field. The participation of one of the researchers in ritual activities and their integration as a teacher in the community facilitated trust-building and access to local information. The conversation begins in Quechua with the expression: *tapuchakusaiki* (can I ask you some questions?). The villager responds: *allinmi, imachataam tapuwanki* (it is fine, what is the question about?). It is then specified: *chay ñaupaq unqukuymanta* (the question is about the ancient custom of childbirth). The interlocutor responds: *allinmi, chayqa kunanqa ancha kanchu* (it is fine, now there is almost none of that). In this way, the dialogue begins, as when questions are direct, the responses tend to be brief and not very extensive.

## 3 Results

### 3.1 Ethnomedical knowledge and perinatal care practices

Ancestral knowledge of childbirth, grounded in empirical practices transmitted across generations, continues to be present in some remote communities of Peru, where the role of traditional midwives remains significant. In contexts characterized by limited access to medical services, families turn to midwives, especially during post-hospital discharge care, a stage in which the mother's and newborn's recovery and care continue within the domestic setting. Their presence, therefore, remains a necessity. This is expressed by an 88-year-old Andean midwife: “I learned from my mother, who was a midwife. From a very young age I accompanied her when she attended women, and by observing and assisting, I gradually acquired the knowledge, since in my community there was no health center.” Various beliefs persist within communities that conceive childbirth as an event of profound familial and communal importance, especially in a contemporary context marked by declining fertility rates. Although social development and the expansion of health services have contributed to a substantial reduction in neonatal mortality, this does not delegitimize the persistence of beliefs and practices associated with *ñaupaq qina unqukuy* (childbirth as in ancient times), which continue to be valued as part of cultural memory and ancestral knowledge of maternal and neonatal care.

Andean communities have their own ways of conceiving pregnancy, childbirth, the postpartum period, and newborn care; thus, “beliefs and customs associated with pregnancy and childbirth have accumulated over time, when a woman is pregnant she participates in a series of beliefs and popular rituals” (10). These rituals, moreover, require the intervention of a specialist. One of the interviewed midwives explains, referring to the incorrect position of the baby during pregnancy: “Then I perform *qaqupa* [rubbing the abdomen] and *suysu* [shaking] with a blanket, up to five months of gestation, to position it correctly.” Pregnant women confirm that this practice helps correct fetal positioning. According to Laureano-Eugenio et al. (11), pregnant women and traditional midwives express “the need for recognition and appreciation of traditional midwifery practices and for providing spaces for their professionalization, proposing the recovery of the “natural origin of childbirth care” and the promotion of “respect for women's dignity”” (p. 280). In the study community, there persists a fear of identifying themselves as midwives due to medical stigma that devalues their work in an activity considered high-risk during childbirth.

The childbirth process in an Andean community has profound social implications, in which older adult women actively participate by supporting the experienced midwife. When asked about the differences between ancestral and contemporary childbirth, one 88-year-old interviewee stated: “According to our knowledge, we use medicinal plants and natural remedies. In modern medicine, chemical drugs are used; if the woman cannot give birth, a cesarean section is performed, and within three or four days she is made to get up. In addition, her diet is no longer cared for as before.” The knowledge transmitted across generations acquires central value in legitimizing these practices, since “traditional health agents are midwives, herbalists or *yuyeros*, and community sages. As members of the community, they have provided ancestral medical services based on experiential knowledge” (12). This perspective recognizes community knowledge but tends to idealize ancestral medicine without problematizing its limitations, scientific validation, or its necessary intercultural articulation with biomedical systems.

Their traditional knowledge proves effective when intervening in childbirth; their experience generates trust and security within the community. When asked about how to improve postpartum health, a midwife explains: “After childbirth, the woman is bound with a *rulliti* and a *chumpi*, and remains inside the house during her recovery. She is bathed with herbs such as *qatqi qura* and *wayra qura*, and medicinal plants are administered to promote internal healing and the gradual recovery of her strength. She is strictly cared for so that she does not drink cold water, is not exposed to drafts, or carry heavy objects, thus avoiding possible relapses. The baby's umbilical cord is dried, fragmented, boiled, crushed, and given to the mother to drink as part of her healing process.” Postpartum care is essential, especially in contexts where there are limitations in access to drinking water, sanitation services, and electricity. In this scenario, “in rural areas, midwives are an important support that allows the health sector to enter the community, as they have the trust of the people and understand the organization and culture surrounding maternity” (13). This highlights their strategic role as intercultural mediators, although it assumes harmonious cooperation without problematizing the structural tensions inherent in the health system.

Newborn care is essential, even in contexts of extreme poverty where living conditions are precarious; nevertheless, childbirth is experienced as a moment of joy. A midwife states regarding neonatal care: “They pray the Our Father five times because the baby has been born well. Then they wrap the baby to protect it from air and cold; afterward, they massage the entire body with hot medicinal plants or also perform incense rituals.” Clothing and bathing of the newborn are fundamental practices carried out inside the home, without the rustic nature of the dwelling being an obstacle. At the same time, there is a certain resistance toward hospital births with medical care, since “in many cultural contexts, childbirth has been considered a moment of great significance, where the woman is connected with the strength of her ancestors” (14). This perspective emphasizes the symbolic dimension and ancestral continuity, although it may essentialize motherhood and overlook the diversity of contemporary female experiences. The main categories are summarized in Tables 1, 2.

**Table 1:** Thematic matrix of ethnomedical knowledge and perinatal care practices.

Central theme	Identified subtheme	Ethnographic interpretation	Representative testimony
*Ñaupa tiempo* (ancient time)	Cultural memory of traditional childbirth	Ancestral childbirth is remembered as a communal practice linked to the experience of ancestors.	“In the past, everyone was born like that in the community.”
*Suysupa* (moving with a blanket)	Traditional adjustment techniques	Midwives use body movements with a blanket to reposition the baby and relieve discomfort.	“With the blanket, the baby is adjusted when something is wrong.”
Medicinal plants	Ancestral therapeutic use	Infusions and herbs continue to be used during pregnancy, childbirth, and postpartum.	“Hot herbs help the mother recover.”
Wrapping (*fajado*)	Neonatal protection and care	Wrapping is understood as a practice of bodily protection and strengthening during the postpartum period.	“The mother must be well wrapped to recover her strength.”

Prepared by the authors.

**Table 2:** Thematic matrix: andean worldview and the spiritual dimension of birth.

Thematic category	Interpretative finding	Empirical evidence	Representative quote
Preservation of customs	Childbirth and maternal care practices are maintained as knowledge transmitted across generations	Persistence of rituals, use of medicinal plants, and continuity of traditional childbirth in rural settings	“We still continue with ñaupaq qina unqukuy”
Voluntary practice	Participation of midwives and community actors is based on voluntary service	Continuous support for pregnant women without institutional remuneration	“By observing, we learned from our mothers and grandmothers”
Godparenthood and umbilical cord	Newborn care includes symbolic practices linked to umbilical cord handling and ritual kinship relations	Participation of godparents in birth rituals and newborn protection	“Childbirth is not only the woman's responsibility; the whole family accompanies it”
Traditional postpartum care	Postpartum care is carried out at home through traditional recovery practices	Use of herbs, massages, and follow-up by midwives after hospital discharge	“When she returns from the health post, the midwife continues caring for her”

Prepared by the authors.

### 3.2 Andean worldview and the spiritual dimension of birth

To understand the experience of ancestral heritage in the Andes, it is necessary to consider that, within the Peruvian social structure—predominantly Quechua-speaking—various ancestral beliefs and practices related to daily life persist: “where the population is predominantly Quechua-speaking, various ancestral beliefs and practices related to daily life persist. These beliefs and the sustained use of traditions strengthen social cohesion among community members” (15). This statement recognizes the cohesive function of culture, although it tends to homogenize the Quechua population and overlooks internal dynamics of change, conflict, and diversity. Cosmovision constitutes a central element, as expressed by the only male midwife in this study: “In our community, these values are part of our customs and are preserved with deep respect. For a woman to have a good childbirth, offerings are made to Pachamama, presenting various products; then it is asked that both the mother and the baby be well and alive.”

Although childbirth is predominantly attended by women, there are exceptions in which some men practice as traditional birth attendants. Regarding the performance of this significant role, a mother states: “Before childbirth, permission was asked from Pachamama so that everything would go well, through a small offering or a prayer. The mother had to take care of herself, avoid cold, and refrain from heavy work. Birth was not only the mother's concern; the entire community participated, practicing *ayni* and helping one another.” Currently, their participation in childbirth processes is less frequent due to the intervention of medical personnel, who at times devalue their work, as well as the decline in fertility rates in the Andes. As a form of compensation, he explains: “They thanked me when the birth went well; they would offer me a gift, such as a guinea pig or potatoes, and in some cases they asked me to be the child's godfather. All of this expresses respect and trust.” This constitutes the main form of compensatory recognition within the Andean community.

Care against illnesses that may affect the newborn and the mother is essential. The case of umbilical cord cutting is particularly significant; as noted in other testimonies, it is roasted and consumed by the mother to prevent illnesses during the postpartum period. Another testimony records an Andean popular belief: “When the baby is born, the first thing done is to cut the umbilical cord with a *kallana* or a broken tile, since it cannot be cut with a knife. When asked why, she responded that if a knife is used, the child quickly wears out their shoes and clothes, and it may also cause bleeding in the navel and lead to infection. She gives an example to her own son, who was cut with a knife and, according to her, indeed wears out his clothing and footwear quickly. The godfather and godmother assume this responsibility with commitment.” Living conditions of poverty in the Andes influence these beliefs related to clothing care, considering that garments are often inherited from older to younger siblings. In relation to these statements, “midwives state that women in rural communities have greater geographical, cultural, and economic difficulties in accessing second- and third-level hospitals than women in urban areas” (11). This highlights structural territorial and sociocultural gaps in maternal health, underscoring a persistent systemic inequality between rural and urban settings.

The use of natural resources from the surrounding environment constitutes a central axis in traditional childbirth and postpartum care. In line with this, the testimony of a midwife, who has also experienced motherhood, reinforces the continuity of this ancestral knowledge: “Postpartum care is based on ancestral knowledge that integrates warmth, nutrition, and bodily regulation. The woman is protected with blankets and bands to prevent the “cooling” of the body, and she consumes *wayra muña* infusion for internal recovery. Nutrition is regulated through solar observation, beginning at the appropriate time with finely chopped and soft meat, according to bodily fragility. These practices are sustained through intergenerational transmission and family support, which ensures the mother's comprehensive recovery.” Together, this forms a coherent postpartum care system in which ancestral knowledge integrates body, nature, and community, reinforcing a holistic conception of health and cultural continuity. The main categories are summarized in the following table:

### 3.3 Oral reproduction of knowledge within the community

Quechua, although it has undergone standardization processes and has official recognition in Peru, transmits traditional knowledge mainly through oral means, in which adults play a central role in its preservation and intergenerational reproduction. This process is embedded in a context of structural tension with modern medicine, although dynamics of articulation between both knowledge systems are also evident. In this regard, a comparative study conducted in another sociocultural context states that “the integration of traditional and modern knowledge is also evident during the childbirth process. Childbirth is no longer permitted solely under the care of traditional midwives (paraji), but must be assisted by obstetricians or specialized healthcare personnel” (16). Maternal and neonatal care practices vary across communities. A midwife states: “After cutting the umbilical cord, it was measured by hand, leaving approximately 10 cm; it was believed that its length influenced the size of the baby's genitalia, both in boys and girls. Then, the baby's body was adjusted and tightly wrapped, in order to promote strong, healthy, and resilient growth.” This reflects a practice based on symbolic bodily beliefs, in which neonatal manipulation is intended to influence physical development and future health.

The advance of biomedicine in Andean territories has generated a process of progressive displacement of traditional childbirth practices, currently recorded mainly through oral accounts from older adult populations. The analysis of testimonies reveals regular patterns in newborn care, particularly in the handling of the umbilical cord. An informant states: “The newborn is not bathed immediately: first it is gently cleaned and then bathed, taking care of its joints. In case of infections, talcum powder or ground *chuño* is used. Proper drying is emphasized to prevent illnesses. Traditionally, the godmother cuts the umbilical cord with a tile or *kallana*, not a knife. Today, this practice has been replaced by care in health centers.” In contexts with limited availability of industrial supplies, the use of *chuño* flour as a substitute for talcum powder is documented, as well as the use of tile fragments for cutting the umbilical cord, associated with symbolic beliefs about future effects on the individual. It is observed that, despite material deficiencies, puerperal care remains a central axis within the domestic sphere: “The care provided to postpartum women remains alive in the intimate space of the home, where knowledge related to female care is reproduced” (17). These findings evidence the persistence of female knowledge systems that structure maternal care practices in local communities.

Mutual support and community experience constitute a central axis in the transmission and practice of ancestral knowledge related to childbirth, the postpartum period, and newborn care, where differentiated practices are articulated within an integrated care system. Field testimonies evidence the empirical effectiveness of ancestral midwifery and its contrast with modern medicine, particularly in the postpartum period, which is traditionally characterized by 45 days of rest and a specific diet based on roasted potatoes, *chuño*, and dried meat (*charqui*), with the exclusion of fresh foods, associated with the recovery of maternal bodily balance. Complementary practices are identified, such as the use of steam baths with medicinal plants, whose relaxing effects contribute to reducing postpartum stress and support maternal recovery: “that various plants used in steam baths by postpartum mothers have a relaxing effect, and therefore can reduce postpartum stress” (18). Taken together, these elements demonstrate coherent knowledge systems regarding maternal care in Andean peoples.

The oral transmission of knowledge among pregnant women constitutes a central mechanism in the continuity of community cultural practices and in the preservation of knowledge associated with maternal and neonatal care. In this intergenerational process, “the modes of transmission, where older women emphasize community-religious meanings and traditional earthen-floor contexts, while younger practitioners increasingly adopt artistic-individual approaches, modern surfaces, and digital forms of learning” (19). This finding highlights transformations in cultural transmission practices, although symbolic elements linked to the Andean worldview persist. In neonatal care practices, ritual mechanisms oriented toward the spiritual protection of the newborn are identified. An older midwife states: “If you travel far, you must use black soot; with it, you should paint the baby's forehead and nose so that the mountain does not see him/her.” This practice expresses the conception of mountains as entities capable of influencing child health and reflects the articulation between territory, spirituality, and ritual protection. Religious differences influence forms of social regulation and cultural legitimization, evidencing tensions and coexistences between traditional practices, Christian beliefs, and biomedical models present in Andean society.

The tension with biomedicine was evident in everyday clinical encounters at the local health center, particularly during prenatal check-ups, institutional childbirth care, and postpartum care. Some mothers reported that traditional practices such as the use of medicinal plants, the wrapping (*fajado*) of the newborn, or the participation of midwives during childbirth were questioned or restricted by health personnel. Quechua-speaking women reported cultural and linguistic communication difficulties when attempting to explain practices associated with *wachakuy*. From the participants' perspective, these situations generated distance and mistrust toward institutional healthcare. A large part of the collected testimonies is retrospective in nature, due to the contemporary decline of traditional ancestral childbirth practices. The gradual incorporation of obstetric and nursing techniques promoted by state health policies has contributed to the partial displacement of traditional childbirth knowledge. Consequently, the participation of midwives tends to be concentrated mainly in postpartum care and newborn attention within community spaces located outside the immediate reach of medical services.

Collective memory among the population evidences the persistence of shared knowledge regarding pregnancy, childbirth, the postpartum period, and newborn care, particularly among women, which configures an intergenerational knowledge system. Ancestral midwifery knowledge was recognized as Intangible Cultural Heritage of Humanity by the United Nations Educational, Scientific and Cultural Organization (UNESCO) in 2023, contributing to its visibility and legitimation as cultural heritage (2). This recognition is articulated with testimonies that demonstrate the continued existence of these practices: “When the baby is born, there are already customs to protect and care for them. The whole family prays and asks God (*Taytacha*) so that the baby may live well, healthy, and in harmony with the entire family. Afterwards, baths and incense rituals with medicinal herbs are performed so that nothing bad affects them: so that wind, fright, or bad air do not harm them. The baby is also tightly wrapped so that cold does not enter. In this way, the newborn is protected from an early age.” These neonatal care practices integrate religious, therapeutic, and symbolic dimensions, articulating protection strategies against environmental and spiritual risks.

### 3.4 Social role and community female leadership

Midwives trained through intergenerational transmission constitute key agents in Andean communities, not only in the health domain but also in the reconfiguration of power relations. Their practice places women at the center of contexts historically marked by male predominance. In this regard, “in Andean communities, marked by the predominance of male power, this is changing substantially with the transfer of power to women. The symbolism of Inca governance, still present today, is being transferred to women in markedly macho contexts” (20). This process evidences a transformation of Andean sociocultural structures, where female empowerment resignifies symbolic inheritances of Incaic origin. At the empirical level, testimonies indicate that traditional midwifery practice is configured as an integrated system of care: it includes prenatal accompaniment, interventions in cases of placental retention, abdominal massage, monitoring of gestational development, guidance during childbirth, and postpartum care. Altogether, these actions consolidate a continuous care model that articulates ancestral knowledge, risk prevention, and comprehensive maternal care.

Midwives hold moral authority in Andean communities to guide and regulate practices related to pregnancy, the puerperium, and neonatal care. This authority is expressed both through the transmission of knowledge and through their capacity to question external practices. In particular, a critical stance is evident toward the contemporary biomedical model, perceived as a process of medicalization of childbirth that displaces traditional practices and weakens culturally significant postpartum care. As an experienced midwife states: “Now they take women to the hospital, they give them injections, IV fluids, and sometimes they cut their abdomen. Before, childbirth was natural and waited for with patience. Women recovered with broths and weeks of rest. Now in the hospital they no longer provide that care or those foods.” In parallel, the historical analysis of gender relations allows these transformations to be situated within a broader scenario of structural tensions. During the colonial period and its continuities, Indigenous women faced pressures associated with the social reproduction of the group, which in multiple cases led to migration processes toward urban spaces.

Field data collection shows that the ancestral childbirth ritual, locally referred to as “natural,” involves the entire family group. However, in response to the sanitary requirements of the Peruvian State and the risk of legal sanctions, pregnant women attend health posts. After returning to their communities, traditional practices related to the puerperium and neonatal care are reactivated. In this case, an elderly midwife states: “During childbirth, the woman walks slowly when the pain begins; as it intensifies, she steadies herself and gathers strength, with the support of her husband. Clothing, leather, or a space on the ground is prepared, since birth takes place at home or in the field, with the help of neighbors. The baby may be born together with the placenta. The placenta is not discarded; it is buried near the hearth, in a well-covered hole to protect it from the wind, following traditional custom.” These findings make it possible to identify a traditional obstetric practice of a community-based nature, structured around the mobility of the pregnant woman, family support, home birth, neighbor participation, and the ritual handling of the placenta, constituting an integrated care system grounded in sociocultural foundations.

The experience of labor in rural societies remains a recurrent practice, associated with limited family planning; in remote areas, households report between six and seven children. Midwives consolidate skills and forms of empowerment linked to their practice. An interview states: “Knowledge is configured as an embodied practice, learned from a young age through observation and participation alongside one's mother and grandmother. During the period of displacement due to violence, where access to health services was nonexistent, childbirth was sustained in the domestic space. There, learning is reinforced through action: accompanying the pregnant woman, physically and emotionally supporting her, and facilitating a safe birth. This knowledge is reproduced within the family and communal sphere, integrating maternal and neonatal care.” From an analytical perspective, the circulation of embodied knowledge across generations in contexts of violence becomes evident, where home birth articulates care, resilience, and community reproduction. It is summarized in Table 3.

**Table 3:** Thematic matrix: social role and community female leadership.

Thematic category	Identified subtheme	Ethnographic interpretation	Representative testimony
Hospital and cesarean section	Institutionalization of childbirth	Childbirth has been progressively shifted toward the hospital setting	“Now more women go to the hospital and fewer call the midwife.”
Neighbor support	Community support networks	Pregnancy and childbirth care is sustained through neighborhood solidarity	“When a child is born, the whole family gathers.”
Companionship during pregnancy	Family and community support	Pregnancy and childbirth occur with the constant presence of relatives and community actors	“They don't always understand how we think in the community.”
Continuity of traditional knowledge	Generational transmission	Knowledge about childbirth is learned through observation and practice, maintaining its validity	“No one taught us from books; we learned by watching.”

Prepared by the authors.

### 3.5 Intercultural asymmetries and processes of delegitimization of local knowledge

A structural disconnect is identified between local Andean culture and modern medical practices, which tend to be imposed in a top-down manner, delegitimizing the ancestral experience linked to the role of the midwife in postpartum and newborn care. This process is intensified by biomedical advancement and institutionalized intercultural approaches, where “although “the cultural” appears as a politically correct discourse and even as a “commonplace” in health professionals' practice, it often does not end up operating in the same way” (21). This reveals a gap between normative intercultural discourse and actual healthcare practice, in which culture is symbolically acknowledged without effective implementation. In this context, an-interview indicates: “Postpartum care involves protecting the woman from wind and cold, considered risks for her recovery. For five days, steam baths are applied to induce sweating and restore bodily balance. In cases of pain, rituals are performed to “open the body” and relieve discomfort, thus guiding a holistic healing process.” This testimony reflects a postpartum therapeutic system based on thermal-body balance, where ritual practices and steam therapies integrate care, prevention, and comprehensive female recovery.

Regulatory frameworks are identified that restrict traditional childbirth practices in Peru, within a social environment where processes of intercultural articulation with biomedicine are insufficient, contributing to the delegitimization of ancestral knowledge. The lack of official records on maternal mortality linked to midwifery care limits comparative analysis. However, the problem persists at a national scale: “maternal mortality in Peru continues to be a public health problem in the country, where the Loreto region accounts for 25% of total deaths nationwide” (22). In analytical terms, this reveals territorial inequalities and structural gaps in access, quality, and coverage of healthcare services. In this context, an-interview states: “It is an ancient knowledge that we have inherited from our grandmothers. It is important because we care for the life of the mother and the baby with respect, using our customs and medicinal plants, so that our culture is not lost.” This testimony reflects the continuity of ancestral female knowledge, where traditional practices and the use of medicinal plants articulate maternal care and cultural preservation.

In the study analyzed, childbirth is conceived as a natural physiological process, managed primarily through outpatient care provided by midwives, which reduces the centrality of biomedical intervention. From the early stages of pregnancy, care practices are implemented aimed at controlling fetal position and monitoring gestational processes, configuring an autonomous care system. Medical intervention is perceived as intrusive and as delegitimizing ancestral knowledge. A midwife states: “The husband takes care of her, bathes her, and helps her change clothes. For five days, she is given hot baths with medicinal herbs, followed by one more week of rest. Gradually, the body is adjusted with a girdle. The baby also receives care until two weeks after birth.” This testimony reveals a postpartum care system based on the family, with defined gender roles, thermal therapies, and rest periods oriented towards maternal recovery and neonatal protection. In contrast, “it is perceived by many women as a natural physiological process, while some interviewees reported experiencing it as a medicalized or even pathological process.”

A process of progressive adaptation of young mothers and couples to biomedical requirements and to the regulatory frameworks of the Peruvian State in matters of health is observed, particularly in response to the problem of maternal mortality. Nevertheless, maternal and neonatal care maintain differentiated local configurations, with shared regularities among communities. In this context, a midwife states: “The newborn is given its first bath with medicinal herbs such as eucalyptus, borage, rosemary, and quichkahuayra. Then it is wrapped warmly and taken to the mother for breastfeeding.” This testimony reflects traditional neonatal practices that integrate the use of medicinal plants, thermal protection, and the early establishment of bonding through breastfeeding. At the same time, persistent forms of resistance and reconfiguration are identified in response to processes of medicalization, understood as “the phenomena of medicalization, but there is no definitive meaning of the concept. What is not in question regarding its definition is that it implies the expansion of medical jurisdiction and its use as a mechanism of social control” (23). Methodologically, this highlights both the conceptual ambiguity of the phenomenon and its expanding role in the regulation of health practices. It is summarized in Table 4.

**Table 4:** Thematic matrix: intercultural asymmetries and delegitimization of local knowledge.

Thematic category	Identified subtheme	Ethnographic interpretation	Representative testimony
Postpartum care of the woman	Postpartum care in the family setting	Care of the woman after childbirth is primarily sustained in the domestic environment, through rest and specific nutrition	“The husband takes care of her, bathes her, and helps her change clothes. For five days, she is given hot baths with medicinal herbs, then one more week of rest.”
Culture is not lost	Continuity and persistence of ancestral knowledge	Cultural practices linked to childbirth and neonatal care do not disappear but remain present in collective memory and everyday practice	“It is an ancient knowledge we have inherited from our grandmothers… this is how our culture is not lost.”
Herbal bath for the newborn	Traditional neonatal care practices	The newborn receives baths with medicinal plants as part of a system of thermal protection and symbolic cleansing	“The baby is given its first bath with medicinal herbs such as eucalyptus, borage, rosemary…”

Prepared by the authors.

## 4 Discussion

The findings allow for the interpretation that ancestral childbirth care practices play a structural role in maternal and neonatal public health, especially in rural environments with limited access to institutional healthcare services. In health crisis situations, such as the COVID-19 pandemic, it became evident that midwives played a critical role not only in rural areas like Huayao but also in urban contexts, facing the collapse of hospital systems and ensuring continuity of maternal care (24). This suggests that traditional knowledge functions as a complementary system of healthcare resilience. The interaction between ancestral practices and contemporary biomedical recommendations generates relevant tensions for maternal and neonatal health indicators. The unsupervised use of traditional medicine among pregnant women may entail potential risks due to the lack of scientific evidence regarding the safety of certain medicinal plants, introducing a dimension of clinical vulnerability (25). This situation directly affects indicators such as maternal morbidity, obstetric complications, and adverse neonatal outcomes.

An institutional interpretation persists that tends to consider the cultural aspects of childbirth as barriers to maternal safety, due to their association with home birth rather than institutional delivery (26). Ethnographic findings show that these practices should not be reduced to risk factors, but rather understood as culturally structured care systems which, when appropriately articulated with the biomedical system, can strengthen service coverage, increase trust in health services, and contribute to the reduction of maternal and neonatal mortality. In contrast to this institutional interpretation, recent evidence during the COVID-19 pandemic reinforces the functional relevance of midwives within health systems. As Melgar Tapia and Yancán Ricaldi (24) note, the pandemic context demonstrated the essential role of midwives not only in rural areas such as Huayao, but also in urban settings, particularly in the face of the collapse of both public and private hospitals. This finding suggests that, far from being a marginal or residual practice, traditional knowledge operates as a complementary and resilient component within maternal care systems, especially in situations of health crisis and structural limitations of biomedical systems.

Knowledge regarding the use of plants in reproductive processes does not constitute an isolated phenomenon but shows cross-cultural correspondence. As documented in other contexts, there is a “female report of cultivated taxa and useful weeds in fertility regulation and for ailments related to menstruation and childbirth… Plants for fertility regulation are often used in secrecy” (27). This finding evidences the existence of specialized female knowledge in managing fertility and reproductive health through plant-based resources, suggesting that these practices are part of complex knowledge systems that integrate health, the body, and the natural environment. It has been noted that “the work at its core is a struggle to keep alive the essential principles of self-care, home remedies, traditional births, and community resilience, which are the main inspirations for the work of Kichwa midwives” (7). This statement highlights processes of cultural resistance and the affirmation of health autonomy in Indigenous societies, where midwives play a central role in sustaining non-institutionalized care practices. From a critical public health perspective, this appreciation must also be problematized, as the idealization of traditional practices may lead to a romanticization of local knowledge without sufficient assessment of its clinical safety, comparative effectiveness, and feasibility within institutionalized health systems.

The findings of this study provide relevant theoretical implications for the field of public health, particularly in relation to the medicalization of childbirth, intercultural mediation, and health equity. Ethnographic evidence confirms that pregnancy and childbirth care in rural and Indigenous societies is not limited to a biomedical model but is articulated with phytotherapeutic and ritual knowledge systems that regulate labor and the puerperium. A systematic use of medicinal plants such as basil, garlic, maidenhair fern, fennel, and rue in oral infusions to accelerate childbirth is observed, as well as chamomile for vaginal washes with anti-inflammatory purposes during the puerperium (28). This expands medicalization theory by showing that biomedicine coexists with local therapeutic rationalities that are not residual, but rather functional within the care system. The results allow for a refinement of classical approaches to the medicalization of childbirth, which tend to interpret traditional practices as deviations from the institutional model. The evidence suggests, on the contrary, that these practices constitute strategies of female agency in response to structural conditions of inequality. It has been documented that “the reaction of Indigenous women to the difficulties of their communities in ensuring the reproduction of the whole group and to the pressures they therefore faced was, on many occasions, migration to cities” (29), which evidences processes of social adaptation that transcend the strictly health domain and involve reproductive and territorial decisions.

This research contributes to theories of intercultural mediation in health by showing that the relationship between biomedical and traditional knowledge is not solely one of conflict, but also of re-signification. This mediation is shaped by symbolic tensions, especially when pregnancy is conceptualized as a pathological process within certain biomedical frameworks. Some studies indicate that “for these individuals, pregnancy had a negative connotation and they wished not to have to experience it as a pathological process” (30), which evidences the internalization of biomedical discourses that may contradict cultural understandings of pregnancy as a natural process. From a health equity perspective, the findings show that the coexistence of differentiated medical systems generates both opportunities and inequalities in access to and quality of care. Overall, this study advances existing theories by proposing that maternal health should be understood as a hybrid field of knowledge, where equity depends not only on institutional coverage but also on the system's capacity to integrate diverse cultural rationalities without delegitimizing them.

## 5 Conclusion

The study's conclusions show that the integration between ancestral knowledge and modern medicine in the field of childbirth is characterized by a progressive deterioration of traditional practices, influenced by the expansion of the biomedical model and the lack of continuity in their transmission. This process is exacerbated by an institutional framework that tends to hold midwives responsible for maternal deaths, generating stigmatization and fear, which leads to the gradual abandonment of their practice. Ancestral knowledge does not disappear completely but is maintained in collective memory and oral transmission in Quechua, where forms of mutual assistance in childbirth and newborn care persist. The practice of natural childbirth continues in rural communities, generally with the support of midwives and experienced women, which evidences the partial persistence of the traditional system in a tense coexistence with modern medicine.

Public health policies should incorporate structural measures for the integration of Andean midwives into the formal health system through institutional recognition, intercultural training, and participation in childbirth care, avoiding their marginalization. Intercultural protocols should be established that include the accompaniment of midwives in health facilities, as well as clear communication regarding biomedical procedures such as cesarean sections, placental management, and umbilical cord cutting. These measures would help reduce distrust toward the health system and improve the articulation between traditional medicine and biomedicine. It is recommended to promote joint training spaces between health personnel and midwives in order to strengthen mutual respect for different knowledge systems, minimizing risks without delegitimizing ancestral knowledge. In doing so, the aim is to preserve the cultural balance perceived by communities and to strengthen equity in maternal healthcare.

Future quantitative research is recommended to measure maternal and neonatal health outcomes in communities where traditional practices and biomedicine coexist, considering indicators such as maternal mortality, obstetric complications, and timely access to health services. Implementation studies on health interculturality policies are needed, aimed at evaluating the articulation between traditional midwives and the biomedical system, especially in geographically hard-to-reach areas. Comparative research on social perception, institutional stigmatization, and the covert use of empirical practices is also suggested in order to understand their impact on maternal safety. It is pertinent to study intercultural training models promoted by the State that enable mutual re-education between health personnel and communities, strengthening the complementarity between ancestral knowledge and modern medicine for the benefit of maternal and child health.

The main limitations of the study are related to the distrust of key actors in narrating their experiences in detail. This attitude is based on the perception that the research could constitute a form of surveillance or devaluation of their beliefs in favor of modern medicine. In addition, there is restricted access to information from mothers whose births were attended by midwives—locally recognized as women experienced in childbirth. During the informed consent process, informants' responses tend to be brief and evasive, expressed in Quechua through phrases such as: “chainallam, manam mastaqa yachanichu” (that is all the information, I do not know more). It is suggested that future research incorporates an intercultural perspective in dialogue with the medical approach, promoting specialized training programs for obstetric emergency care in home settings. This would allow clinical safety to be articulated with the warmth of the family environment, avoiding the stigmatization of deeply rooted cultural practices and fostering care that respects local knowledge systems.

## Data Availability

The original contributions presented in the study are included in the article/Supplementary Material, further inquiries can be directed to the corresponding author.
