# Supplementary material for: Rituals and knowledge of Andean midwives in maternal and neonatal care
Source: Front Glob Womens Health. 2026 Jul 14;7:1857301. doi: 10.3389/fgwh.2026.1857301 (PMC13408237; doi:10.3389/fgwh.2026.1857301)
Supplement: Supplementary file 1 [file Datasheet1.pdf]

Supplementary Material

1 Supplementary Data

INTERVIEW 1
Name: Mrs. Nathalia (name changed for confidentiality)
Age: 63 years
Occupation: Peasant farmer
Place: San Miguel Community - Patibanba
Topic: Good evening, Mama Nathalia, my name is Yolanda, a student from the private higher pedagogical school CLAM, how are you? Good, Miss Yolanda. Yes, I would like you to tell me about your experience as a midwife in your community. As we were already talking, could you please tell me what is called ancestral wisdom of Andean midwives in the care of the mother and the newborn? Well, Mrs. Nathalia told me in her mother tongue, which is the Quechua of her community; we were able to establish a fluent conversation with Mrs. Nathalia.

1.- What is ancestral midwifery and what is its importance in the Andean community?
She helps her child and the mother when the time of giving birth arrives, to pull; before that, they place a small cloth on the stomach called *rulliti*, and on top of that they tie an Andean belt called *chumpi*. After the baby is born, they lay them on the bed. We keep the custom alive in our community so that it does not disappear. Since when have you been a midwife? I knew I had a baby in 1963 and I knew it that year.

2.- What functions does the midwife fulfill during pregnancy, childbirth, and postpartum?
The midwife accompanies the woman from the first months of pregnancy. When the placenta does not come out, she helps it come out by giving oil or covering the belly with egg white. She also gives massages on the belly to position the baby and relieve discomfort. She advises not to carry heavy things and to take care of oneself from the moment the pregnancy is noticed. From five months on, the midwife observes how the belly is growing and prepares the mother for childbirth.
During childbirth, she takes care that the woman does not get cold, does not sit or stand up abruptly, and guides her so that the birth is natural. After childbirth, the midwife continues caring for the mother to prevent illnesses and help her recovery.

3.- How is the knowledge of ancestral midwifery transmitted from one generation to another?
I learned by watching my mother. Observing how she did her work, that is how I learned. In that way I understood and now I know how to do it. It was not in school, but by watching and doing.

4.- What role do spiritual and cultural values play in the practice of midwifery?
The woman is protected from wind and cold. For five days she is made to sweat with vapors. Rituals are also performed when there is pain, because in that way the body opens and relief comes. These care practices help the whole body to heal.

5.- What differences exist between ancestral midwife care and modern medical care?
Before everything was better. Now they take women to the hospital, give them injections, IV fluids, and sometimes cut their belly. Before, childbirth was natural and awaited with patience. Women

recovered with broths and rest for weeks. Now in the hospital they no longer receive that care or those foods.

6.- What recommendations do midwives provide during pregnancy?
The midwife advises not to do heavy work, to eat well, to walk, and not to stay sitting all the time. If she does not take care, the baby can grow improperly. All this is so that childbirth is easier.

7.- How do they prepare the mother for the moment of childbirth?
Yes, she is advised like this: “do passes, move carefully.” She is made to walk slowly, to walk and walk every time the pains come. When the pains become stronger, the woman begins to prepare herself, to steady herself well and gather strength for childbirth. The husband also supports her and holds her to give her strength. For the moment of childbirth, clothing or a hide is prepared, or also a space on the ground. Everything must be ready. Before, in the field or at home, right there the baby was born. Sometimes the woman gave birth on a skin, and when a neighbor arrived, she helped. In the corral or in the house the baby was born. Sometimes the baby came out together with the placenta and then everything was arranged. Was the placenta thrown away before like they do now in hospitals when the woman gives birth?
No, daughter, it was not like that. Before it was buried next to the kitchen hearth. Why was it buried near the hearth? Because there a hole was made in the ground and it was buried, to protect it from the wind. That is how it was done, covering it well. If there was wind, the door was closed. That is how the placenta was treated. We do not know if now in hospitals they do something similar or what they do.

8.- What practices are carried out after childbirth for the recovery of the woman?
Yes, the husband takes good care of her, attends to her and is in charge of her. He helps her bathe and change clothes. For five days she is given hot baths with all the medicinal herbs, and then she must rest for one more week. Then, in a third stage, little by little her body is tightened again with the belt, carefully. Even so, she must continue taking care not to do heavy work and not to get cold, keeping warm and eating properly. She still should not make great efforts. The baby is also bathed, and in this way both mother and child are cared for for approximately two weeks. After that time, the mother can bathe normally. During all this period, the baby is protected from cold and illness, ensuring well-being from the beginning of life.

9.- What care does the baby receive immediately after birth?
That is, one must only take care so that the baby does not get sick. The baby must be protected from wind, sun, cold, fright, the moon, and the stars. And what happens if the baby looks at the sun or the moon? The baby becomes crybaby and weak. When that happens, how is it cured? With dry herbs, the baby is smoked, kept warm, and given heat. If there is wind, the hearth is lit to make smoke, and there the baby is smoked. Even until the baby chokes a little with the smoke, the baby is smoked, because that is good so that it heals. That is how the baby is cared for from the URIWAS.

10.- What rituals or customs are performed to protect the newborn?
If you travel far with black *ollen*, you must paint the baby’s forehead and nose so that the mountain does not look at him/her.

11.- What meaning does the first bath or the swaddling of the newborn have in the Andean tradition?

When the newborn baby is bathed, it is not done immediately, because the baby still has a bad smell and remains on the little body; first it is gently cleaned and then bathed, also taking care of the joints. If pus were to appear, with what would it be cured? Talc or ground *chuño* is used; first it is applied to the affected part and with that it heals. That is why I have never made my baby get sick; even when I bathed the baby, I always dried him/her well. Also, to cut the umbilical cord, the godmother was sought to do it. Before it was cut with tile or *kallana*, because that was considered better.
And why could the baby's umbilical cord not be cut with a knife?
Yes, I had cut my child's then his clothes and his shoes finish quickly, they do not last long. From before, the godmother cut the umbilical cord with tile or *kallana*. Now the comadre is almost no longer recognized, because in the health post the doctors cut the umbilical cord, whether the obstetrician or another person.
Then at the moment of cutting the umbilical cord it was cut measuring with the hand about 10 centimeters; if you cut smaller, they say that the baby's penis is also small, and if you cut bigger, it is also big, and in the same way if it were a girl. Then the baby's little body is arranged well, and so that the baby sleeps well, it is swaddled, wrapped and tied well; in that way the baby grows strong, healthy, and with more resistance.

12.- How does ancestral midwifery strengthen the bond between the mother, the baby, and the community?
Thus, from the moment the mother becomes pregnant, she is cared for and accompanied, spoken to and advised until the day of childbirth arrives. The midwife cares for the mother and the baby from the first moment of pregnancy; from then on the mother speaks to her belly, caresses it and rubs it gently, and by doing so, the baby moves inside the womb and begins to recognize. In this way, the community also becomes involved: when the mother becomes ill, they come with food, with products from the field, with potatoes, meat or other supplies, and they also help by cleaning the house or caring for the baby or the mother. Thus they live well, practicing *ayni* (mutual help), supporting each other and living together in harmony

INTERVIEW 2
Name: Mrs. Greta (name changed for confidentiality)
Age: 88 years
Occupation: Peasant farmer
Place: Andahualas Community - Pakucha
Topic: Good morning Mrs. Greta, my name is Yolanda Justina Soliz Yaranga, student of the private higher pedagogical education school CLAM. First of all, I would like to thank you for your time. My research topic is "Ancestral wisdom of Andean midwives in the care of the mother and the newborn," which aims to understand and value. Well, that is fine Miss Yolanda, I will gladly answer your questions.

1.- What is ancestral midwifery and what is its importance in the Andean community?
Midwifery is the care that is done as if she were an obstetrician, given to pregnant mothers, and at the moment of giving birth they take care of them, providing all the necessary care.
Since when have you practiced the work of a midwife? Well, I have practiced for many years. I learned from my grandparents and mothers by observing and accompanying them in births. Since then, I have carried out this work with much respect with the women of the Pakucha community.

2.- What functions does the midwife fulfill during pregnancy, childbirth, and postpartum?
During pregnancy, instructions are also given on how she should take care of herself: not lifting weight, not staying seated but always walking so that the fetus does not develop too much.

3.- How is the knowledge of ancestral midwifery transmitted from one generation to another?
Through the experience that I have had from my ancestors.

4.- What role do spiritual and cultural values play in the practice of midwifery?
If they are believers, they have to comply with all the mechanisms that give blessing. And socially, they also have to adapt to customs, depending on belief. In this case we can see perhaps evangelicals with Catholics, but they do not do it very differently, it does not match. The beliefs and also the customs.

5.- What differences exist between ancestral midwife care and modern medical care?
Ancestral midwifery, despite not having as much knowledge, not having the studies that current ones have, we performed very well. Now, nowadays doctors and modern medical care, the main difference would be that before, for the mother who had given birth, she had to stay in bed, rest, without doing work, nothing, for 45 days. But now, only on the second day, she is already going out. In addition, they were given specialized food, which nowadays they give them anything. What kind of food was given in those times? For example, they could not consume boiled potatoes, but had to be roasted. Then *chuño*, dehydrated potatoes. Then, regarding fresh meat, they should not eat it but rather *charqui*. Why should they eat that? Because if they eat fresh things, since the woman's body is delicate, it would not agree.

6.- What recommendations do midwives provide during pregnancy?
During pregnancy, they should always be walking. Never stay seated for a long time. Nor lift weights. Why? Because the child, when they are seated and develops too much, becomes chubby and will have difficulty in childbirth.

7.- How do they prepare the mother for the moment of childbirth?
They prepare her with walks and massages on the back and holding the waist each time labor pains come, giving her hot infusions for rapid dilation such as chamomile, anise, and others.

8.- What practices are carried out after childbirth for the recovery of the woman?
She must rest for 45 days, without touching water or lifting weight, and must feed herself with broths to produce milk. In addition, she must wash with aromatic herbs.

9.- What care does the baby receive immediately after birth?
The newborn, the first bath that is given is with medicinal herbs, aromatic herbs so that the baby does not get sick, so that the air does not affect them. And what kind of herbs could it be? Eucalyptus, borage, and rosemary; another herb is *quichkahuayra*. Then the baby is wrapped warmly and taken to the mother so that she can breastfeed.

10.- What rituals or customs are performed to protect the newborn?
Well, prayers or fumigations with herbs are performed to ward off bad energies. Amulets with red thread were also placed so that no one casts the evil eye, thus protecting the newborn.

11.- What meaning does the first bath or the swaddling of the newborn have in the Andean tradition?
First, the umbilical cord is cut. In the past they did not use a blade but tile, a piece of tile or glass. Then the umbilical cord is tied by making a knot, and then cotton is placed on top of that, and a coin. And what happens if it is not tied? It can have something like, it will cry, it will strain. It can have a hernia.
So that the navel does not come out, after the bath, sheep *millwa* is placed in a round shape on the head, and that makes the little hollow warm so that cold does not enter. Besides that, the baby's body when it is born has like a white cream, like grease; that white grease the midwives remove very carefully, and that little cream should be used by the mother who developed spots during pregnancy; with that cream her face is cleaned. Meanwhile, swaddling is so that the body develops correctly, so that the arms and legs are not bowed.

12.- How does ancestral midwifery strengthen the bond between the mother, the baby, and the community?
Well, it strengthens from the moment she becomes pregnant, through the care she receives at the time of childbirth, and constant accompaniment at all times until the baby is born, giving advice and help. The baby is also spoken to, cared for from cold and fright, and in that way there is a unique connection between mother and baby. With the community as well, when a baby is born it is a unique joy, where they come with food or clothes or help the mother for her recovery.

INTERVIEW 3

Name: Manuel (name changed for confidentiality)
Age: 64 years old
Occupation: Farmer
Location: Sacharaccay Community – Atoqwachanqa

Good morning, Mr. Manuel. My name is Yolanda, a student at the Private Higher Pedagogical Education School CLAM. How are you?

I am fine, Yolanda. Now, I will answer your questions. You previously asked me about illnesses and how I have treated women, and also my own mother. Therefore, I will share this information with you under the title:
"Ancestral Wisdom of Andean Midwives in the Care of the Mother and the Newborn."

I was born in 1961, truthfully. Thank you, Mr. Manuel, now let us begin.

1. What is ancestral midwifery and what is its importance in the Andean community?

For us, ancestral midwifery is something very valuable and sacred, because from the moment women become pregnant, they are cared for and accompanied so that the baby is born healthy. In our community, midwifery is highly valued and respected by both women and men.

Why did a man have to be a midwife?
Because men have more physical strength. When a woman could not endure the pain or effort, the

man would help by holding her firmly and massaging her abdomen to support her and prevent illness. That is why men were sought to help in those difficult moments.

Since when have you practiced midwifery?
Since I was very young. First, I assisted my mother when she became ill. I was born in 1961, and from the age of 7 or 8 I observed my uncles, relatives, and grandparents attending births. I kept all that in my mind, and that is how I learned.

2. What functions does the midwife fulfill during pregnancy, childbirth, and postpartum?

First, when a woman becomes pregnant, she is given broths and nutritious foods to strengthen her, because the baby grows inside her womb and needs proper nourishment.

In the past, pregnant women were carefully cared for. Elders advised that whether the baby was male or female, the mother should maintain strength and good spirit. They prepared chuño broth, thick soups, and gave her abundant food in large bowls with wooden spoons, unlike today.

How did you assist in midwifery?
Pregnancy lasts nine months. When labor began, if the baby was not properly positioned, I would say: "Do not worry, I know how to help you." Then I would ask for a blanket (lliklla) and carefully reposition the baby so it would not be transverse, as that could lead to death.

Once properly positioned, the woman felt less pain and became calmer.

As labor approached, all necessary materials were prepared: ropes, belts (chumpi), blankets, and remedies. Herbal baths were given using wayra muña, kichka qura, and small muña. The woman's head was also wrapped to give her strength.

After the baby was born, the mother was carefully assisted to expel the placenta, lifting her gently and burying it properly.

What happens if the placenta is not removed quickly?
If it remains inside, the mother's condition may worsen. Therefore, it must be removed and buried properly, keeping the environment protected from cold air.

Afterward, her diet is carefully managed, and she is kept well covered.

How should the mother be fed after giving birth?
While still delicate, she is given chuño soup and thick broth with mint and dried meat (charqui), to prevent body pain and cold. Food should not be too hot or too cold. If too hot, the baby may develop diarrhea; if too cold, illness may occur.

It is said that when a female baby is born, the mother recovers faster (about three months), whereas with a male baby, recovery may take three to four months.

Male babies are considered stronger and move more in the womb, which can weaken the mother during labor. Therefore, both mother and baby require special care, proper nutrition, and warmth.

3. How is this knowledge transmitted?

I learned by observing my family, my grandfather and grandmother. By watching and listening, I retained this knowledge from childhood.

4. What role do spiritual and cultural values play?

In our community, these values are part of our traditions and are respected. Offerings are made to Pachamama with different products, asking for the well-being and life of both mother and baby.

5. Differences between ancestral and modern medical care

We treat using medicinal plants and natural remedies. Modern medicine uses chemical drugs, and when a woman cannot give birth, surgery (cesarean section) is performed, and she is made to get up after a few days. Also, nutrition is not always cared for as before.

6. Recommendations during pregnancy

Women are advised to avoid heavy labor, not carry heavy loads, and eat well. Near delivery, they should not wear sandals (ojotas).

Why not sandals?
Because the baby is already positioned, and improper movement could affect the womb or baby. Instead, they used leather footwear (siquy) and covered their heads with a cloth.

How was siquy made?
It was made from sheep or goat leather, dried and carefully prepared for firm walking.

7. Preparation for childbirth

Before, there were no beds. Wooden structures were built and covered with sheepskins and woven blankets.

During labor, a wide cloth and belts (chumpi) were tied around the abdomen to stabilize the baby's position.

Medicinal herbs prepared included olorosa, mint, marqarinqa, pacha taya, qillqimsa, turunqil, and kichka flowers.

These herbs protect against cold, wind, earth, and water, helping the mother recover strength. Some grow in mountains or rocks (like alakasa).

They may be dried for three days or used fresh but always clean.

The midwife works with family support. Steam baths are given before birth, and herbal infusions (waramuña, tutawayra, rosemary) are given afterward.

If the woman cannot give birth, alternative remedies are used. One remedy involved an animal (kuqaqwaman), prepared with alcohol and applied or ingested in small amounts, which helped in a difficult case.

8. Postpartum recovery practices

The mother is wrapped warmly with blankets and belts. She drinks wayra muña infusion.

Food is introduced gradually, starting with finely chopped meat. Timing was determined using the position of the sun's shadow.

Soft food is necessary because the body is still delicate.

9. Care of the newborn

The umbilical cord is cut using a ceramic fragment (kallana), not a knife or scissors. A longer portion is left so the baby grows strong.

Cotton and a coin are placed on the navel and secured with a belt to aid healing.

10. Protection of the newborn

Prayers are offered to God (Taytacha). Herbal baths and smoke rituals protect against wind, fright, and harmful air.

Herbs used include:

- Rue (protection from fear)
- Muña (warmth)
- Rosemary (cleansing)

11. Meaning of the first bath and swaddling

The baby is bathed with warm water, blessed with the sign of the cross, and lightly given water orally.

Afterward, the baby is swaddled to keep the body straight and protected.

After three days, the godmother arrives to bathe, dress, and care for the baby, including covering the ears to protect from wind.

12. How does ancestral midwifery strengthen bonds?

From pregnancy onward, the mother and baby bond through touch and voice.

The community supports the mother with food and help, practicing **ayni** (mutual aid), strengthening social ties and collective well-being.

INTERVIEW 4

Name: Mrs. Graciela (name changed for confidentiality)
Age: 90 years old
Occupation: Farmer
Location: Vinchos – Anchaqwasi
Topic: Ancestral Wisdom of Andean Midwives in the Care of the Mother and the Newborn

1. What is ancestral midwifery and what is its importance in the Andean community?

It is the traditional practice of accompanying women during pregnancy, childbirth, and the postpartum period, based on knowledge passed down from generation to generation. Its importance lies in preserving the health of the mother and the baby, strengthening family and community bonds, and maintaining the cultural and spiritual values of the Andean region.

2. What functions does the midwife fulfill during pregnancy, childbirth, and postpartum?

From the moment it is known that a woman is pregnant, the midwife cares for and accompanies her, massaging her abdomen and speaking to her, observing whether the baby is well positioned or sideways, and advising her so she can have a good delivery.

When labor begins, the midwife assists her and stays with her until the baby is born. After childbirth, she continues supporting her and teaches her how to properly care for the baby.

3. How is this knowledge transmitted from one generation to another?

Yes, I learned from my mother, and my mother told me she learned from her grandmother. By observing, she stored that knowledge in her mind, and I learned in the same way—not from books, but from experience and practice.

4. What role do spiritual and cultural values play in midwifery?

For us, spiritual and cultural values are very important. We believe that wind can make the body ill, so we perform steam baths to protect and care for ourselves. This care is given for five days so that the woman regains strength and emotional balance.

When there is pain, the body "opens" and weakens, so it must be carefully restored with respect and faith, so the woman can recover her strength and remain healthy and balanced.

5. What differences exist between ancestral midwifery care and modern medical care?

Now things are no longer as before. From my perspective, everything used to be better. Today, women go to the hospital, receive injections, sometimes become sicker, or even have their abdomen cut (cesarean section). That is how it is done now, but it was not like that before.

In the past, births were natural, and pain was endured calmly. Now, they are quickly taken away, and within one or two days complications may arise.

In contrast, in the countryside, women were cared for by their husbands and families for two to three weeks. They were given warm broth and appropriate food. Now they are not even given broth; I do not know what food they are given in hospitals.

6. What recommendations do midwives provide during pregnancy?

They say that women should eat well so their milk flows properly and the baby can be healthy. If milk does not come out, they are given cooked cow intestine or quinoa soup.

They are also advised to rest, avoid heavy work, avoid cold, not become frightened, and drink medicinal herbs. In addition, they are told not to worry or feel sadness.

7. How is the mother prepared for childbirth?

First, the midwife speaks to the mother and gently massages her abdomen. Then her abdomen is washed with warm water and wrapped with a cloth or tied with a wide belt to keep the baby in position. She is also spoken to so she does not feel afraid.

The mother holds onto a rope, and when contractions come, she pushes with effort and courage to give birth. If the baby cannot come out, the midwife helps with her hands by repositioning the baby.

After birth, the placenta is properly buried to prevent air from entering, as this could cause swelling.

8. What practices are carried out after childbirth for the woman's recovery?

After giving birth, the woman is wrapped with a cloth (rulliti) and a belt (chumpi). She is bathed with herbs such as qatqi qura and wayra qura, always inside the house.

She is also given medicinal plants to heal internally so she can regain her strength over time. She is cared for so she does not drink cold water, get chilled, or carry heavy loads, as this could cause a relapse.

If a relapse occurs, how is it treated?
It is treated using the baby's dried umbilical cord. It is dried, cut into small pieces, boiled in water, then crushed until soft and given to the mother to drink. With this, she recovers.

9. What care does the newborn receive immediately after birth?

They pray the "Our Father" five times so that the baby has been born well. Then the baby is wrapped to protect it from air and cold, and the entire body is massaged with warm medicinal plants or fumigated with herbal smoke.

10. What rituals or customs are performed to protect the newborn?

The baby is fumigated with herbs to protect it from harmful air. Sometimes a red thread is tied to prevent fear.

Additionally, the baby is dressed in yellow clothing for good luck. In some cases, when traveling far, the baby is wrapped in red clothing to avoid encountering the mountains (spiritual harm).

11. What is the significance of the first bath or swaddling in Andean tradition?

When the baby is born, the umbilical cord is cut with a clean, sharpened piece of tile. A coin is placed on the navel and secured with a belt (chumpi).

Then the baby is given its first bath with warm water at a warm time of day. This is done so the baby grows strong, well-formed, with straight limbs and good energy.

12. How does ancestral midwifery strengthen the bond between mother, baby, and community?

From the moment a woman becomes pregnant, she is cared for, observed, and spoken to until the day of birth. The midwife speaks to both the mother and the baby from the beginning, gently touching and massaging the abdomen, which calms the baby.

Likewise, the community participates: when someone is ill, everyone brings food, clothing, or meat. They visit each other, clean the house, and help both mother and baby. In this way, they live well by practicing mutual aid (**ayni**) and supporting one another in solidarity.

INTERVIEW 5

Name: Mrs. Sara (name changed for confidentiality)
Age: 70 years old
Occupation: Farmer
Location: San Miguel – Chilinqa

1. What is ancestral midwifery and what is its importance in the Andean community?

For me, ancestral midwifery is an ancient body of knowledge that we have inherited from our grandmothers. It is important because we care for the lives of both the mother and the baby with respect, using our customs and medicinal plants, ensuring that our culture is preserved.

2. What functions does the midwife fulfill during pregnancy, childbirth, and postpartum?

I accompany the woman from the moment she is pregnant, I examine her, and I give her advice. During childbirth, I assist her with patience and care. After birth, I take care of both the mother and the baby until they become strong.

3. How is this knowledge transmitted from one generation to another?

I learned this knowledge by observing and helping my mother and other elder midwives. That is how it is transmitted—from generation to generation—through practice and respect.

4. What role do spiritual and cultural values play in midwifery?

They play a very important role because everything we do is done with faith and respect for Pachamama and the Apus. We pray and ask for protection for both the mother and the baby.

5. What differences exist between ancestral midwifery care and modern medical care?

The difference is that I provide care with closeness and affection; I know the family and their customs. Modern medicine uses machines and doctors. Both can help, but our knowledge is more closely connected to the community.

6. What recommendations do midwives provide during pregnancy?

I recommend eating well, not carrying heavy loads, staying warm, resting, and avoiding fear. I also advise drinking herbal infusions and remaining calm.

7. How is the mother prepared for childbirth?

I prepare her with massages, warm herbal infusions, warm baths, and encouraging words so that she does not feel afraid and trusts her body.

8. What practices are carried out after childbirth for the woman's recovery?

After childbirth, I apply abdominal binding, give her warm herbal infusions, keep her well covered, and advise her to rest so she can regain her strength.

9. What care does the newborn receive immediately after birth?

I clean the baby (wawa), keep the baby warm, care for the umbilical cord, and ensure that the baby breastfeeds soon so that they grow strong and healthy.

10. What rituals or customs are performed to protect the newborn?

Prayers and herbal fumigations are performed, and sometimes a bracelet or protective object is placed on the baby to protect against harmful air and fright.

11. What is the significance of the first bath or swaddling in the Andean tradition?

The first bath is to cleanse and protect the baby, and swaddling helps the baby's body grow straight and strong, as taught by the elders.

12. How does ancestral midwifery strengthen the bond between mother, baby, and community?

Midwifery strengthens this bond because everyone supports the mother. The baby is born surrounded by affection, support, and traditions, and in this way the community comes together to protect life.

INTERVIEW No. 6

Interviewee: Mrs. Nathalia (name changed for confidentiality)
Location: San Miguel Community – Patibanba

Yolanda: Good evening, Ms. Nathalia. My name is Yolanda, a student at the CLAM Private Higher Pedagogical School. How are you?

Nathalia: I am well, Ms. Yolanda. Yes, I would like to share my experience as a midwife in the community.

Yolanda: As we were discussing, could you explain what the ancestral wisdom of Andean midwives means in caring for the mother and the newborn?

Nathalia: Well, I learned it in my Quechua language. The midwife helps the mother and the baby during childbirth. First, she places a small cloth on the stomach, called rulliti, and on top of that an Andean belt called chumpi. After the baby is born, the baby is laid on the bed. We keep this custom alive so that it does not disappear.

Yolanda: Since when have you been a midwife?

Nathalia: I knew I had to help women since 1963, when my first child was born.

Yolanda: What functions does the midwife fulfill during pregnancy, childbirth, and postpartum?

Nathalia: During pregnancy, she accompanies the woman, gives massages to position the baby and relieve discomfort, advises her not to carry heavy things, helps the placenta come out using oils or egg white, and observes how the belly grows. During childbirth, she ensures that the mother does not get cold or move abruptly, and guides her toward a natural birth. After childbirth, she cares for the mother's recovery with hot baths, rest, proper nutrition, and body adjustment using the belt. She also protects the baby.

Yolanda: How is this knowledge transmitted from one generation to another?

Nathalia: I learned by observing my mother and watching how she worked. It is not learned in school, but through practice and observation.

Yolanda: What role do spiritual and cultural values play in midwifery?

Nathalia: We protect the mother from wind and cold, we carry out sweat baths for five days, and rituals to relieve pain, so the body opens and heals.

Yolanda: What differences do you see between midwifery care and modern medical care?

Nathalia: Before, births were natural, with patience and prolonged care. Now, in the hospital, they use injections, IV fluids, or cesarean sections, without providing traditional care or special foods.

Yolanda: What recommendations does the midwife provide during pregnancy?

Nathalia: That she should not do heavy work, that she should eat well, walk, and not remain seated for too long. All this helps make childbirth easier and the baby grow healthy.

Yolanda: How is the mother prepared for childbirth?

Nathalia: She is advised to walk slowly and move carefully as the pains begin. The husband supports and holds the woman. Clothes, leather, or a space on the ground are prepared. Sometimes women used to give birth on an animal hide at home or in the field. The placenta was buried near the hearth to protect it from the wind.

Yolanda: What practices are carried out after childbirth for the mother's recovery?

Nathalia: The husband takes care of her, bathes her, and helps her change. For five days she receives hot baths with medicinal herbs, then rests for another week. Gradually, the body is adjusted with the belt. The baby also receives care until two weeks after birth.

Yolanda: What care does the baby receive immediately after birth?

Nathalia: The baby is protected from cold, wind, sun, fright, the moon, and the stars. The baby is fumigated with smoke and kept warm. If pus appears on the baby's body, powder or ground chuno is applied.

Yolanda: What rituals or customs are performed to protect the newborn?

Nathalia: If traveling far, the baby's forehead and nose are marked with black ollen so that the mountain does not see the baby.

Yolanda: What is the significance of the first bath and the baby's swaddling?

Nathalia: The baby is not bathed immediately; first, it is gently cleaned. The godmother cuts the umbilical cord with a tile or kallana, and the swaddling wraps the baby so that it grows strong and healthy.

Yolanda: How does midwifery strengthen the bond between mother, baby, and community?

Nathalia: From pregnancy, the mother is cared for and accompanied. The baby recognizes the mother's voice and affection. The community helps with food, cleaning, and care, practicing ayni (mutual aid) and living together in harmony.

Researcher's Notes (post-interview reflection)

Today, after concluding the interview with Mrs. Nathalia, I feel deeply grateful for the opportunity to listen to and document the ancestral wisdom of our community. Her account not only allowed me to understand the techniques and care that Andean midwives apply during pregnancy, childbirth, and postpartum, but also to appreciate the spiritual and cultural dimension embedded in these practices. I was struck by how the entire process is integrated into the family and community environment. The way the mother, baby, husband, and community participate demonstrates a model of collective care based on mutual aid, respect, and harmony. Practices such as fumigation, herbal baths, newborn swaddling, and the burial of the placenta show that ancestral midwifery combines practical knowledge with symbolic elements that protect and strengthen life.

In addition, I observed that the transmission of this knowledge does not take place in a classroom, but through observation and constant practice. Mrs. Nathalia learned by watching her mother, and today she continues this legacy, ensuring that new generations know and respect these traditions. This led me to reflect on the difference with modern medical care: although hospital medicine offers safety and technology, some of these traditional practices could complement current care, especially emotional support, patience during childbirth, and extended postpartum care.

Finally, this interview reminded me of the importance of documenting and valuing ancestral knowledge, not only as technical knowledge but as a living cultural heritage that strengthens community identity and fosters emotional bonds between mother, child, and society.

INTERVIEW No. 7

Interviewee: Mrs. Greta, 88 years old, farmer
Location: Community of Andahuaylas – Pakucha

Yolanda: Good morning, Mrs. Greta. My name is Yolanda Justina Soliz Yaranga, a student at the CLAM Private Higher Pedagogical School. First, I would like to thank you for your time. My research is about the "Ancestral wisdom of Andean midwives in the care of the mother and the newborn." Is it okay if I ask you some questions?

Greta: Good morning, Ms. Yolanda. I will gladly answer your questions.

Yolanda: What is ancestral midwifery and what is its importance in the Andean community?

Greta: Midwifery is the care provided to pregnant women and at the time of childbirth, taking care of them and the baby, providing all necessary care. It is very important because it supports the health of mothers and newborns.

Yolanda: Since when have you practiced as a midwife?

Greta: I have practiced for many years. I learned from my grandparents and mothers, by observing and accompanying them during births. Since then, I have worked with respect in the Pakucha community.

Yolanda: What functions does the midwife fulfill during pregnancy, childbirth, and postpartum?

Greta: During pregnancy, guidance is given on how to take care of oneself: not lifting heavy loads, walking regularly, and not sitting for too long so that the fetus does not grow excessively. During childbirth, we provide massages on the back and waist and give warm infusions for faster dilation. After childbirth, the mother must rest, eat broths, and wash with aromatic herbs.

Yolanda: How is this knowledge transmitted from generation to generation?

Greta: It is transmitted through the experience we have had with our ancestors, by observing and accompanying births.

Yolanda: What role do spiritual and cultural values play in midwifery practice?

Greta: They are very important. Believers follow blessing practices and adapt to the customs of the community, depending on their beliefs, whether Catholic or Evangelical.

Yolanda: How does ancestral midwifery differ from modern medical care?

Greta: Although ancestral midwifery does not have formal studies, we perform very well. In the past, the mother had to rest for 45 days with specific foods such as roasted potatoes, chuno, or charqui. Today, with modern medicine, the mother is discharged on the second day and the diet is less controlled.

Yolanda: What recommendations does the midwife provide during pregnancy?

Greta: To always walk, not sit for too long, and not lift heavy loads, so that the baby does not have difficulties during childbirth.

Yolanda: How is the mother prepared for childbirth?

Greta: With walking, massages on the back and waist, and warm infusions such as chamomile or anise to help dilation.

Yolanda: What care is provided after childbirth for the mother's recovery?

Greta: The mother rests for 45 days, does not lift heavy loads, eats broths, washes with aromatic herbs, and takes care of milk production.

Yolanda: What care does the baby receive immediately after birth?

Greta: The first bath is given with medicinal herbs such as eucalyptus, borage, rosemary, and quichkahuayra. Then the baby is kept warm and taken to the mother for breastfeeding.

Yolanda: What rituals or customs are performed to protect the newborn?

Greta: Prayers and fumigations are done to ward off negative energies, and amulets such as red threads are placed to protect against the evil eye.

Yolanda: What is the significance of the first bath or swaddling in the Andean tradition?

Greta: The umbilical cord is cut with a piece of tile or glass and tied with cotton and a coin to prevent hernias. Then "Millwa," sheep wool, is placed on the head to protect from the cold. The baby's white fat is used to clean the mother's face, and the swaddling helps the baby's body develop properly.

Yolanda: How does ancestral midwifery strengthen the bond between mother, baby, and community?

Greta: The midwife accompanies the mother from pregnancy, providing advice and care. The community celebrates the birth with food, clothing, and support, strengthening the bonds among everyone.

Researcher's Notes (post-interview reflection)

The interview with Mrs. Greta allowed me to deeply understand the richness of ancestral wisdom in Andean midwifery. Despite her advanced age, her memory and clarity in describing the processes of pregnancy, childbirth, and postpartum are remarkable. I was struck by how her knowledge is based not only on practical experience but also on respect for the cultural and spiritual values of the community.

I observed that ancestral midwifery is not limited to physical procedures, but also integrates emotional and social care, strengthening the bond between mother, baby, and community. Mrs. Greta emphasized the importance of specific nutrition, prolonged rest, and the use of medicinal herbs, as well as protective rituals for the newborn, reflecting the Andean worldview of health and balance.

One aspect that impressed me was how ancestral midwifery knowledge is transmitted from generation to generation not through books or formal education, but through observation, continuous practice, and oral transmission, highlighting the importance of preserving this tradition in the face of the modernization of medical care.

Finally, I reflected on the importance of valuing and documenting this knowledge. The interview showed that ancestral midwifery not only fulfills a healthcare function but also reinforces social cohesion and cultural identity within the Andean community. This experience motivated me to recognize the need to respect and preserve these practices, which combine knowledge, spirituality, and humanity.

INTERVIEW No. 8

Interviewee: Mr. Manuel, 64 years old, farmer
Location: Community of Sacharaccay

Yolanda: Good morning, Mr. Manuel. I am Yolanda. As I mentioned, I am conducting research on the ancestral wisdom of Andean midwives in the care of mothers and newborns. I would like you to share your experience and knowledge.

Mr. Manuel: Yes, Yolanda. I know many things that I learned by observing and helping from a young age, following what my mother and grandmother taught me. During the time of terrorism, we had to move to Ayacucho with our families, and there was no access to medical care. At that time, women gave birth at home, and I helped by observing and assisting in everything I could. I learned how to care for the mother during childbirth, how to help her stay strong, how to calm her, and how to support the baby's healthy birth. I even helped my wife when she gave birth, making sure she rested and did not do heavy work. I also taught other women in the community how to care for their newborns.

Yolanda: Mr. Manuel, how can a man become a midwife in your community?

Mr. Manuel: It is not impossible for a man. It requires willingness, observation, and practice. I learned by watching my mother and grandmother and helping women when they had difficulties. When the mother could not push enough, we would gently massage her abdomen with warm oil, carefully positioning the baby. Everything is done with respect and patience.

Yolanda: In your community, did husbands mind if you helped during childbirth?

Mr. Manuel: No, on the contrary, they were grateful. When the birth went well, they would offer me a gift such as a guinea pig or potatoes, and sometimes they asked me to be the child's godfather. This shows respect and trust.

Yolanda: Did you use medicinal plants during childbirth and after birth?

Mr. Manuel: Yes. We used herbs such as muña to prepare infusions or steam for the mother to inhale, protecting her from the cold and helping her recovery. We also gave hot herbal baths to the mother and the baby, and we paid special attention to the umbilical cord to prevent infections. Every detail had its purpose.

Yolanda: Were there spiritual or cultural care practices or recommendations?

Mr. Manuel: Yes. Before childbirth, we asked permission from Pachamama so that everything would go well. A small offering was placed or a prayer was said. The mother had to protect herself from the cold and avoid heavy work. Birth was not only about the mother; the whole community participated, practicing ayni, helping one another.

Yolanda: Thank you very much, Mr. Manuel, for sharing your knowledge and experience.

Mr. Manuel: Thank you, daughter, for taking an interest in preserving what we know. I hope these teachings are not lost.

Researcher's Notes (post-interview reflection)

Mr. Manuel demonstrated deep knowledge of Andean ancestral midwifery, transmitted orally from generation to generation. Midwifery is not limited to childbirth, but integrates physical, emotional, cultural, and spiritual care, as well as the use of medicinal plants and traditional techniques to protect both mother and baby. It was highlighted that community practice and cooperation (ayni) are

fundamental, and that the role of men as midwives, although less common, is possible within the tradition.

The interview confirms that ancestral midwifery remains necessary in rural areas with limited access to healthcare services, and that this knowledge should be recognized, valued, and preserved as part of the Andean cultural heritage.

INTERVIEW No. 9

Unstructured Interview

Interviewee: Mrs. Graciela, 98 years old, farmer
Location: Vinchos – Anchaqwasi

Yolanda: Mrs. Graciela, what is ancestral midwifery and why is it important in our community?

Graciela: It is the traditional way of accompanying a woman from the moment she becomes pregnant until after childbirth. It is important because it cares for the health of both the mother and the baby, preserves our customs, and strengthens the family and the community.

Yolanda: What does the midwife do during pregnancy, childbirth, and postpartum?

Graciela: During pregnancy, we care for her, speak to the baby, massage her, and give advice so that the birth goes well. During childbirth, we assist her until the baby is born, and afterward we teach her how to care for the child and accompany her so she recovers well.

Yolanda: How did you learn all of this?

Graciela: I learned from my mother, and she from her grandmother. It is learned by observing, practicing, and keeping the knowledge in memory, not in books.

Yolanda: What role do cultural and spiritual values play?

Graciela: They are very important. We perform steam baths to protect from the wind, and we rely on faith and respect so that the mother regains her strength.

Yolanda: What differences do you see between midwifery care and modern hospital care?

Graciela: Before, births were normal and calm, and the mother was cared for at home with food and family support. Now, in hospitals, sometimes they make them sicker, give injections, or cut the belly.

Yolanda: What recommendations are given during pregnancy?

Graciela: To eat well, rest, not work too much, avoid cold and fear, take medicinal herbs, and remain calm.

Yolanda: How is the mother prepared for childbirth?

Graciela: She is spoken to, her abdomen is massaged, she is washed with warm water, a belt is placed so the baby does not move, and when the pains come, the mother pushes while the midwife helps if the baby cannot come out.

Yolanda: And after childbirth, what care does the mother receive?

Graciela: She is wrapped with a chumpi, bathed with herbs, given medicinal plants, and must not carry heavy things or drink cold water. If there is a relapse, we use traditional remedies with the baby's umbilical cord.

Yolanda: What care does the newborn receive?

Graciela: Prayers are said, the baby is kept warm, massaged with warm plants, and fumigated to protect from air and illness.

Yolanda: What is the meaning of the baby's swaddling and first bath?

Graciela: They help the baby grow strong and balanced. The umbilical cord is cut with a clean tile, a coin is placed, and the first bath is given with warm water.

Yolanda: How does ancestral midwifery help strengthen the bond between the mother, baby, and community?

Graciela: From pregnancy, the mother receives care and words from the midwife, and the community participates by helping, sharing food, and offering support. In this way, everyone lives in solidarity and harmony.

Researcher's Notes (post-interview reflection)

After the interview with Mrs. Graciela, I feel deeply impressed by the richness of the ancestral knowledge she has transmitted throughout her life. Her clarity in describing care during pregnancy, childbirth, and postpartum demonstrates that midwifery is not only a medical practice but also a fabric of cultural, spiritual, and community values that strengthen the bonds between mother, baby, and community.

I was struck by how rituals and practices such as steam baths, fumigations, and newborn swaddling not only have a physical meaning but also aim to harmonize the body, mind, and spirit, preserving emotional and social health. Mrs. Graciela repeatedly emphasized the importance of patience, respect, and faith, aspects that I consider essential for understanding midwifery as an integral form of knowledge beyond the technical dimension.

Reflecting further, I also perceived the tension between modern medicine and tradition. Mrs. Graciela described with nostalgia the care of the past, when childbirth was a natural process supported by the family, compared to the rapid and sometimes impersonal interventions in hospitals. This highlights

the importance of documenting and preserving these practices, as they represent not only care techniques but also cultural identity and collective memory.

Finally, the interview reminded me that ancestral midwifery is an example of how practical and spiritual knowledge can sustain a community. The constant interaction between the mother, the midwife, and the community reflects an approach of mutual support and solidarity, known as ayni, which is essential for understanding health and well-being in rural Andean contexts. This experience motivates me to continue researching and disseminating this knowledge, always respecting the voices of those who keep it alive.

INTERVIEW No. 10

Interviewee: Mrs. Sara, 70 years old, farmer
Location: San Miguel – Chilinqa

Yolanda: Mrs. Sara, what is ancestral midwifery and why is it important for the community?

Sara: Ancestral midwifery is knowledge that was taught to us by our grandmothers. It is important because we care for the lives of the mother and the baby with respect, using our customs and medicinal plants. In this way, our culture is preserved.

Yolanda: What functions does the midwife fulfill during pregnancy, childbirth, and postpartum?

Sara: I accompany the woman from the moment she is pregnant, I examine her, and I give her advice. During childbirth, I help her give birth with patience and care. After childbirth, I take care of both the mother and the baby until they become strong.

Yolanda: How does a midwife learn this knowledge?

Sara: I learned by observing and helping my mother and other elder midwives. This is how it is transmitted, from generation to generation, through practice and respect.

Yolanda: What role do spiritual and cultural values play in your work?

Sara: Everything we do is done with faith, respecting Pachamama and the Apus. We pray and ask for protection for the mother and the baby because the spiritual aspect is very important.

Yolanda: How does ancestral midwifery differ from modern medical care?

Sara: The midwife provides care with closeness and affection and knows the family and their customs. Modern medicine uses machines and doctors. Both help, but our knowledge is closer to the community.

Yolanda: What recommendations do you give during pregnancy?

Sara: To eat well, rest, stay warm, not carry heavy loads, and not be frightened. I also recommend drinking herbal infusions and staying calm.

Yolanda: How do you prepare the mother for childbirth?

Sara: With massages, warm herbal infusions, warm baths, and encouraging words so she is not afraid and trusts her body.

Yolanda: What practices do you carry out after childbirth for the mother's recovery?

Sara: I apply abdominal binding, give her warm herbal infusions, keep her warm, and tell her to rest to regain her strength.

Yolanda: What care does the newborn receive?

Sara: I clean the baby, keep the baby warm, care for the umbilical cord, and ensure that the baby breastfeeds soon so that they become strong and healthy.

Yolanda: What rituals or customs do you perform to protect the newborn?

Sara: We perform prayers and fumigations, and sometimes we place a bracelet or a protective object to protect the baby from harmful air and fright.

Yolanda: What is the significance of the first bath and the baby's swaddling?

Sara: The first bath is to clean and protect the baby. Swaddling helps the baby's body grow straight and strong, as taught by the elders.

Yolanda: Finally, how does ancestral midwifery strengthen the bond between the mother, baby, and the community?

Sara: Midwifery strengthens the bond because everyone supports the mother. The baby is born surrounded by affection, support, and traditions, and in this way the community comes together to protect life.

Researcher's Notes (post-interview reflection)

The interview with Mrs. Sara made it possible to understand the importance of ancestral midwifery in the Andean community. She expressed pride in the knowledge inherited from her grandmothers, which combines the physical care of the mother and baby with spiritual and cultural values. It stands out that the midwife uses medicinal plants, rituals, and prayers, safeguarding health while providing emotional security to the mother.

In addition, her work strengthens family and community bonds, as everyone participates in the care surrounding birth. Ancestral midwifery differs from modern medicine in its closeness and knowledge of the family context, although both can complement each other. Overall, this practice not only protects life but also preserves culture and reinforces community cohesion.

INTERVIEW No. 11
Location: San Miguel Community – Patibamba

I had a prior conversation with Ms. Nathalia one week earlier, and on December 11, 2025, she asked me to travel to the city of Ayacucho to visit her children. I arrived on the agreed date and time, five minutes early, and Ms. Nathalia was already waiting for me. She received me very kindly. At 6:30 p.m., we began our conversation.

Ms. Nathalia shared her experience regarding the "ancestral knowledge of Andean midwives in the care of mothers and newborns." She explained that she learned to become a midwife when she had her first child; at that moment, she felt called to help women in her community. She stated that when a woman becomes pregnant, she should not lift heavy objects. Before giving birth, a ritual known as **PAQAPU** must be performed, using fruits, coca leaves, candles, sweets, liquor, or **PACHA QAMPI**, and a live guinea pig. The guinea pig is passed over the pregnant woman's body, and then, around midnight, she is taken with two companions to the designated place.

For childbirth, a rope must be prepared and tied to a strong beam of the house, known in Quechua as **KUMBLIRA**, along with a goatskin or sheepskin and plastic to receive the baby. When the baby is born, the umbilical cord is cut using a **KALLANA** or a broken roof tile, not a knife. When I asked why, she explained that using a knife causes excessive bleeding and may lead to infection. She provided a personal example, stating that her own child's cord was cut with a knife, and the child's clothes and shoes wore out quickly, which is culturally interpreted as a negative outcome. The godfather and godmother assume responsibility for this act.

After birth, the baby is bathed with warm water. Once dried, the umbilical cord is wrapped with a binder, and the baby's arms and legs are straightened and wrapped with a white cloth so that the child sleeps well, grows strong, and rests longer.

She further explained that during pregnancy, up to five months, it is important to monitor the baby's position. If necessary, a technique called **SUYSUPA** is performed using a blanket, with great care, to ensure proper positioning for childbirth. For postpartum recovery, the mother must consume beef or chicken broth to promote breast milk production. Medicinal herbs are also used internally and externally for recovery.

Regarding newborn care, she emphasized preventive practices to avoid illness, including the concept of **URIWA** (exposure to sun or stars). If the baby is affected, the child is fumigated with smoke from the **tullpa** (traditional stove) until a mild reaction occurs, which is believed to cure the condition. Additionally, baby clothes left outside after 4:30 p.m. must be brought inside; otherwise, the baby may suffer from **QAYQA**, causing urine to appear like beaten egg. In such cases, the juice of ripe **ÑUCHKU** fruit is given to the baby for healing.

Finally, she expressed her opinion about hospital care today, stating that she does not agree with it. She indicated that when women experience labor pain and cannot give birth, hospitals often perform cesarean sections and administer injections, while providing insufficient nutrition. In contrast, traditional practices rely on medicinal herbs, proper nutrition, and patience, without surgical intervention. She emphasized that she learned midwifery from her mother. The interview was conducted primarily in her native language, Quechua, and lasted until 9:10 p.m.

Researcher's Reflection

During the observation, I was able to appreciate the richness and depth of the ancestral knowledge of Andean midwives, transmitted from generation to generation through experience and community practice. Ms. Nathalia demonstrates that being a midwife involves not only technical knowledge about pregnancy and childbirth, but also a social and ethical commitment to the community, guiding and supporting women at critical moments in their lives.

I was particularly struck by how each practice serves a specific purpose, addressing the physical, emotional, and spiritual health of both mother and newborn. Rituals such as **PAQAPU**, **SUYSUPA**, fumigation with **tullpa**, and umbilical cord care using **kallana** or a broken tile aim not only to protect the baby's health but also to create a harmonious bond among mother, child, and family.

The use of medicinal herbs, nutritious food, and specialized newborn care reflects deep knowledge of disease prevention, postpartum recovery, and holistic well-being. I also observed the importance of the Quechua language in transmitting this knowledge, reinforcing cultural identity and ensuring the accuracy and respect of traditional practices.

Furthermore, I reflected on the differences between modern and traditional medicine. Ms. Nathalia indicated that hospital care often does not respect the natural timing of childbirth and prioritizes invasive procedures such as cesarean sections, whereas ancestral medicine values patience, herbal treatments, and proper nutrition to facilitate safe and natural births. This highlights the need to integrate both knowledge systems, promoting respectful, safe, and culturally sensitive care.

The work of Andean midwives goes beyond technical practice; it represents a comprehensive care system that combines empirical knowledge, spirituality, ethics, and social responsibility. This experience allowed me to understand that ancestral knowledge is an invaluable source of identity, cohesion, and health for Andean communities, and its preservation is essential for the well-being of mothers, newborns, and families.

Emergent Questions

Interviewer (Yolanda): Ms. Nathalia, how did you learn to become a midwife?
Interviewee (Nathalia): I learned when I had my first child. At that moment, I realized I could help other women in my community, caring for both mother and baby. My mother taught me all the practices and rituals before, during, and after childbirth.

Interviewer: When a woman becomes pregnant, what care should she follow from the beginning?
Interviewee: From the moment she knows she is pregnant, she should not lift heavy objects or make excessive effort. She is also prepared for childbirth rituals and taught how to care for her body and the baby.

Interviewer: What is PAQAPU and how is it performed?
Interviewee: PAQAPU is a ceremony performed before childbirth. It includes fruits, coca leaves, candles, sweets, liquor, and a live guinea pig. The guinea pig is passed over the pregnant woman's body for protection, and then she is taken with two companions to the designated place. This ensures a safe and harmonious birth.

Interviewer: Why is the umbilical cord cut with a kallana or broken tile instead of a knife?
Interviewee: Because using a knife can cause bleeding and infection. Using a kallana or broken tile is considered safer. Also, the godfather and godmother take responsibility for cutting the cord.

Interviewer: What other care does the newborn receive?
Interviewee: The baby is bathed with warm water, the umbilical cord is wrapped, and the arms and legs are straightened and wrapped with a white cloth so the baby sleeps well and grows strong. We also perform URIWA; if the baby is affected by sun or stars, fumigation with tullpa smoke is used for protection.

Interviewer: What recommendations do you give to the mother after childbirth?
Interviewee: The mother should eat well, especially beef or chicken broth, to produce enough breast milk. Medicinal herbs are also used for recovery, both internally and externally. Continuous care is essential so the baby remains healthy and strong.

Interviewer: What do you think about hospital care nowadays?
Interviewee: Nowadays, hospitals often perform cesarean sections and use medications when women cannot give birth, and nutrition is poor. Before, we relied on herbs, good nutrition, and patience. I believe our ancestral medicine has great value and should be respected.

Interviewer: What is the most important value of being a midwife?
Interviewee: The most important thing is protecting the life of both mother and baby, transmitting ancestral knowledge, preserving cultural identity, and supporting families during such an important moment.

INTERVIEW No. 13
Location: Andahuaylas Community – Pakucha

Ms. Greta is a neighbor who lives near my home. Although she does not currently reside permanently in Ayacucho, she frequently travels to her hometown. I had previously informed her about my research project and invited her to my home to conduct an interview. She kindly accepted and was willing to share her knowledge and experiences as an ancestral midwife, a role she has performed while assisting many women in her community.

The interview began at 5:45 p.m. I offered her a seat so we could speak comfortably in a calm environment. From the beginning, she appeared composed and confident. Ms. Greta is an ancestral midwife with more than 30 years of experience. She is characterized by her calmness, patience, and deep respect for cultural traditions.

When asked how she learned to become a midwife, she stated that she acquired her knowledge from her grandparents through observation and practice. She explained that care for women begins from the moment pregnancy is confirmed. During this stage, she provides guidance on the importance of walking, avoiding heavy lifting, and maintaining appropriate habits to ensure a safe delivery. She also applies ancestral knowledge to guide mothers during childbirth.

She performs back and waist massages to relieve pain, prepares warm herbal infusions such as chamomile and anise to facilitate dilation, and provides constant support to instill confidence. She noted that, in the past, the umbilical cord was cut using a **kallana** or a broken roof tile, a traditional practice in her community.

During the postpartum period, she ensures that the mother rests, generally for 45 days, and maintains a specific diet based on nutritious broths. She also cares for the newborn: she gives the first bath using medicinal herbs, ensures the baby is kept warm, and protects the umbilical cord with cotton and a coin. In addition, she places protective amulets such as red threads to guard the baby against the "evil eye," known as **WAYRURU**.

Ms. Greta also performs rituals such as fumigations and blessings, integrating spiritual and cultural values at every stage of pregnancy, childbirth, and postpartum care. Her relationship with the community is close; she not only attends to mothers individually but also involves family members and neighbors, strengthening solidarity and collective support.

Her experience and knowledge inspire trust and confidence among women and their families. Her practice reflects a balance between physical, emotional, and spiritual care. The conversation concluded at 7:00 p.m. The dialogue was fluid and respectful. We spoke primarily in Spanish, her native language, although she also mentioned some expressions in Quechua, her second language.

Researcher's Reflection

The interview with Ms. Greta allowed me to understand that ancestral midwifery is not merely a practice of pregnancy and childbirth care, but a comprehensive body of knowledge that integrates physical, emotional, and spiritual dimensions. Through her account, it became evident that her learning does not come from formal institutions, but from intergenerational transmission, particularly from her grandparents, highlighting the importance of oral tradition and experiential knowledge in preserving these practices.

I was struck by the confidence and calmness with which she explained each procedure, reflecting the trust she has built within her community over the years. Her support does not end with childbirth; it begins during pregnancy and continues throughout the postpartum period, strengthening the bond between mother, baby, and family environment. This demonstrates that ancestral midwifery also fulfills a social and community function.

It is also important to reflect on the coexistence between ancestral midwifery and modern medicine. Although their approaches differ, both aim to ensure the well-being of the mother and newborn. Ms. Greta's experience demonstrates that these traditional forms of knowledge remain relevant and hold significant cultural value that deserves respect and recognition.

Finally, this experience allowed me to appreciate the cultural richness of my community and to understand that ancestral midwifery not only preserves life, but also sustains identity, beliefs, and spirituality. As a researcher, I reaffirm the importance of documenting and disseminating this knowledge to prevent its loss over time.

Emergent Questions

Interviewer (Yolanda): Good afternoon, Ms. Greta. Thank you very much for accepting my invitation to speak with me about your experience as an ancestral midwife.
Ms. Greta: Good afternoon, dear. Thank you for inviting me. I am happy to share what I have learned.

Interviewer: To begin, could you tell me how you learned to become a midwife?
Ms. Greta: I learned from my grandparents. Since I was young, I accompanied them and observed how they cared for women. Little by little, I learned by watching and practicing. In the past, there were no hospitals nearby, so midwives were very important in the community.

Interviewer: From what moment do you accompany a pregnant woman?
Ms. Greta: From the moment she knows she is pregnant. I explain that she should walk, avoid lifting heavy objects, and take good care of herself. I also recommend that she not sit for long periods and that she eat well so her baby is born healthy.

Interviewer: What do you do during childbirth?
Ms. Greta: During childbirth, I stay with the mother at all times. I give her massages on the back and waist to relieve pain. I also prepare warm infusions, such as chamomile or anise, to help with dilation. The most important thing is to give her confidence and peace of mind.

Interviewer: How was the umbilical cord cut in the past?
Ms. Greta: We used a kallana or a broken roof tile. It was an old custom from our grandparents. Then the umbilical cord was tied properly and protected with cotton and a small coin.

Interviewer: What care do you recommend after childbirth?
Ms. Greta: The mother should rest for at least 45 days and avoid lifting heavy objects. She should drink nutritious broths and bathe with aromatic herbs. This helps her body recover.

Interviewer: And what care is given to the newborn?
Ms. Greta: The baby is given the first bath with medicinal herbs, kept warm, and should receive immediate breastfeeding. We also place a red thread or a wayruru to protect against the evil eye.

Interviewer: Do you perform any ritual or spiritual practice?
Ms. Greta: Yes, we perform fumigations and prayers, depending on the family’s beliefs. We always ask for blessings for the mother and baby.

Interviewer: What is your relationship with the community like?
Ms. Greta: It is good. Families trust me because they have known me for many years. I not only care for the mother but also talk with the family and provide guidance.

Interviewer: Finally, what does being a midwife mean to you?
Ms. Greta: For me, it is a gift and a responsibility. It means helping bring life into the world and caring for the mother and baby with respect and love.

INTERVIEW No. 14
Location: Sacharaccay Community

The observation was conducted at Mr. Manuel's residence in Ayacucho. I arrived 10 minutes early, and he kindly received me, showing willingness to answer my questions and share his ancestral knowledge regarding the care of mothers and newborns.

We began the interview at 10:30 a.m. During the conversation, he recounted his experiences and explained how he learned to become a midwife, confidently describing the traditional practices he inherited from his grandparents. He also shared an experience from a trip to the Amazon region, where he adopted an additional method to assist women during complicated labor. This method involved the use of a small preserved reptile. I also observed his use of simple language and cultural expressions characteristic of his community, as he is a Quechua speaker.

We maintained a meaningful connection throughout the interview until 11:30 a.m., when we shared a small meal of *pusp* with cheese. Afterward, Mr. Manuel began to chew coca leaves (*chakchar*), and we resumed the conversation from 11:45 a.m. until 12:30 p.m. He explained that he learned from family members such as his grandparents, uncles, and aunts. He emphasized that no one formally taught him; rather, he learned through observation and internalization, which allowed him to develop his knowledge of midwifery. Through this experience, he has assisted many women in his community.

Researcher's Reflection

During the observation, I was able to understand the significant importance of ancestral midwifery within the community, as it encompasses not only the physical care of the mother and newborn, but also spiritual and cultural dimensions. Mr. Manuel demonstrated confidence and wisdom while sharing his knowledge, as well as his ability to incorporate practices learned from another community into his own.

These practices, transmitted across generations, include rituals that strengthen the bond between the mother, the newborn, and the community, fostering mutual support during such a meaningful stage as childbirth.

Emergent Questions

Interviewer: Mr. Manuel, who taught you midwifery knowledge, and how did you begin practicing it?
Interviewee: I learned from my grandparents. They were midwives in the community, and since I was young, they took me along when there were births. At first, I only observed; later, they taught me how to examine the abdomen and position the baby. It is not something learned quickly—it takes time and respect.

Interviewer: At what age did you assist your first birth?
Interviewee: I was about 20 years old. My grandfather was with me, guiding me and telling me what to do. I was nervous, but I trusted what I had learned. After that, I continued assisting in other births.

Interviewer: You mentioned that you learned another technique in the Amazon. Could you explain what it consists of?
Interviewee: Yes. When I traveled to the Amazon, I met a healer who taught me a different way to

help when labor becomes complicated. He explained the use of a small preserved reptile, which is rubbed over the woman's body—especially in cases related to *URIWA*. A small piece may also be placed in a warm herbal infusion nearby as a symbol of strength and agility, so that the baby can position itself better. It is not only the object, but also the faith and concentration involved at that moment.

Interviewer: When a woman has difficulties during labor, how do you decide what to do first?
Interviewee: First, I observe and palpate the abdomen to understand the baby's position. I also listen to the mother. Sometimes the problem is fear or shock, so it is important to calm her, speak to her, and give her confidence. If necessary, I use medicinal plants or perform massages to reposition the baby, sometimes using a blanket in a technique known as *SUYSU*, done carefully, and tying a cloth around the head.

Interviewer: According to tradition, how is the newborn cared for after birth?
Interviewee: The baby's umbilical cord is cut with a **kallana** or a tile, leaving approximately 10 cm, which is believed to influence future physical development. Then the baby is bathed with medicinal herbs to prevent exposure to harmful air conditions such as *wayra muña* or *tuta wayra*. The baby is then wrapped well to keep warm. The mother is also carefully attended, because if she becomes weak, the baby may also become ill. The placenta is buried inside the house with doors closed to prevent exposure to air, which could otherwise cause swelling in the mother's body.

Interviewer: Are these forms of knowledge being lost, or are there young people interested in learning?
Interviewee: Nowadays, young people are less interested; they prefer to move to the city. However, some still ask questions. I always say that this knowledge should not be lost because it is part of our culture.

Interviewer: What care do you consider most important for a mother during pregnancy?
Interviewee: The most important thing is to care for the mother's body. She must have good nutrition, avoid excessive weight, walk according to her ability, and maintain emotional calm. Some medicinal herbs are also used to strengthen her.

INTERVIEW No. 15
Location: Vinchos – Anchaqwasi

The interview was conducted on January 8, 2025, at the home of Grandmother Graciela, in the community of Anchaqwasi. It began at 2:00 p.m. and concluded at 4:00 p.m., with an approximate duration of two hours. Grandmother Graciela received us kindly and was willing to share her knowledge.

The interview was conducted in the presence of my research partner; we had previously organized the process. I was responsible for asking the questions, while my partner supported the audio recording using a cellphone as a data collection instrument. The conversation was conducted primarily in Quechua, her native language, which allowed her to express herself more naturally and in greater depth.

During the dialogue, she explained how she accompanies pregnant women from the early stages of pregnancy, performing traditional assessments by palpating the abdomen to verify the baby's

position. If the baby is in an incorrect position, she performs **QAQUPA** and **SUYSU** using a blanket, up to the fifth month of pregnancy. She also emphasized the importance of speaking encouraging words to both the mother and the baby to provide emotional reassurance.

Regarding the newborn, she described that immediately after birth, prayers of gratitude and protection are performed. The baby is then carefully wrapped to prevent exposure to “air” or cold, which could affect the baby’s health. Warm medicinal herbal rubbing and fumigation are also carried out as forms of spiritual protection. She indicated that the umbilical cord is cut using a clean, sharp roof tile. A coin is then placed over the navel before wrapping it with a **chumpi**, with the purpose of ensuring that the baby grows strong and well-formed. The first bath is given with warm water at an appropriate time of day, aiming to strengthen the baby’s body and energy.

The interview concluded at 4:00 p.m., with sincere appreciation expressed for the time and trust provided by Grandmother Graciela.

Researcher’s Reflection

The interview with Grandmother Graciela was a very meaningful experience for me. It allowed me to understand that the work of midwives goes beyond attending childbirth; it involves accompanying the mother from pregnancy through the postnatal period. I was particularly struck by the fact that all her practices carry not only physical but also spiritual significance.

For her, newborn care includes prayers, protection against cold and “air,” the use of medicinal herbs, and the wrapping of the baby with a **chumpi**. This demonstrates that, within the community, health is understood in a holistic manner. It was also significant that the interview was conducted in Quechua, her native language, as it enabled her to express herself more effectively. This led me to reflect on how ancestral knowledge is deeply connected to language and culture.

This experience strengthens my research, as it allows me to further value the knowledge of Andean midwives and recognize that these practices continue to hold importance within the community.

Emergent Questions

Researcher (Yolanda): Grandmother, how did you learn the techniques of QAQUPA and SUYSU?
Grandmother Graciela: Since I was a child, my mother and grandmother taught me. These are forms of knowledge passed down from generation to generation.

Researcher: What is the meaning of placing a coin and using the chumpi on the newborn?
Grandmother Graciela: The coin and the chumpi help the child grow strong and healthy. It is a tradition that protects the body and energy.

Researcher: When do you begin caring for a pregnant woman?
Grandmother Graciela: From the early months, I check how the baby is positioned in the womb and perform abdominal rubbing to ensure proper positioning.

Researcher: What do you do if the baby is in an incorrect position?
Grandmother Graciela: I perform QAQUPA and SUYSU with a blanket, up to the fifth month of pregnancy, to reposition the baby.

Researcher: How do younger mothers perceive these practices?
Grandmother Graciela: Many young women respect and follow them; others prefer to go to the hospital, but they still listen to our advice.

Researcher: What importance do prayers and fumigation have after birth?
Grandmother Graciela: They protect the baby from illness and evil spirits; they are very important for the baby's life and health.

Researcher: How is this knowledge transmitted to new generations?
Grandmother Graciela: We teach daughters, granddaughters, and anyone who wants to learn, showing them step by step, always with respect and care.

Researcher: What do you do if there is a complication during pregnancy or childbirth?
Grandmother Graciela: First, I rely on my experience and traditional care; if necessary, we take the mother to a health center, because life is the most important thing.

INTERVIEW No. 16
Location: San Miguel – Chilinqa

Upon arriving at Ms. Sara's home, she stated that she had no objection to sharing her knowledge and experiences. I was received with a warm smile and a cordial greeting. She invited me into the patio of her home, where we began the conversation.

During the interview, she explained that in her youth she practiced ancestral midwifery, following the teachings passed down by her mother approximately 30 years ago, when she still lived in her community of origin. She stated that she learned out of necessity, since there was no health post in her village and access to a medical center was very distant. Transportation was limited (donkey, horse, or mule), which made it essential to have a midwife in order to avoid risking the lives of the mother and the baby. She indicated that she had the support of the community.

In prenatal care, she recommended that pregnant women—especially first-time mothers—avoid carrying heavy loads to prevent miscarriage and maintain proper nutrition. Women who already had two or more children tended to prepare in advance, as they were already familiar with the necessary care for a safe birth.

During labor, she guided the woman to push calmly, while the husband supported her by holding her waist to give strength during each contraction. If the husband was not present, the woman would hold onto a rope to assist with the effort.

After birth, the umbilical cord was cut with a piece of glass, wrapped in cotton, and carefully monitored to prevent infection. She indicated that when bathing the newborn, water should not enter the navel; it was cleaned with warm water until it fully healed.

Among protective rituals, she mentioned that the tip of the baby's nose was painted red so that the child would "have a straight gaze." Likewise, the newborn was carefully swaddled with a *chumpi* (Andean belt) to keep the arms and legs straight. The baby rested in a hammock (*amaka*), which was previously made from blankets, wooden poles, and ropes tied to posts inside the home.

Regarding postpartum care for the mother, she recommended not carrying water or heavy loads for at least three weeks and remaining on bed rest. She believed that otherwise the mother could become ill and the baby could develop "flu" or cough. To stimulate breast milk production, she prepared broth made from sheep or cow intestines, as well as fennel infusions. She indicated that guinea pig broth should not be consumed, as it was believed to affect future fertility (related to "uriwa").

She also stated that a pregnant woman should not carry a newborn, as it could affect a future delivery. Likewise, a woman who was menstruating should not hold the baby, as it was believed that the baby's skin color could change.

Regarding spiritual protection, she mentioned that when attending a wake, *rue* or *molle* leaves should be placed in the baby's armpit to prevent the effects of "qayqa" (spiritual harm or negative energy).

Finally, she explained that after migrating to the city of Ayacucho, she stopped practicing as a midwife. Additionally, upon adopting the Christian faith, she reconsidered some traditional practices, such as offerings (*paqapus*) and certain smudging rituals, as she believed they were not aligned with her new beliefs.

Researcher's Reflection

Ms. Sara's account allowed me to understand more deeply that ancestral midwifery not only emerged as a response to the absence of health services, but also represented a comprehensive system of care built upon experience, observation, and the Andean worldview. It was not limited to attending childbirth, but involved accompanying the woman before, during, and after birth, considering her physical, emotional, and spiritual well-being.

The practices described reflect a holistic view of maternal and child health. Recommendations regarding nutrition, rest, avoiding heavy lifting, and newborn care demonstrate practical knowledge aimed at protecting life. At the same time, protective rituals highlight the importance of cultural beliefs in understanding the birth process. In this context, care extended beyond the physical body to include protection from negative energies or spiritual influences.

It is also evident that knowledge was transmitted across generations, primarily from mother to daughter, strengthening cultural identity and community belonging. The husband's participation during childbirth demonstrates that birth was a collective event in which the family played an active supportive role.

On the other hand, migration to the city of Ayacucho and the adoption of the Christian faith influenced the transformation of these practices. Some rituals were abandoned as they were considered incompatible with new religious beliefs, reflecting a process of cultural change. However, many practical aspects of maternal and newborn care continue to be valued.

This experience leads me to reflect on the importance of recognizing and valuing ancestral knowledge within current health systems. It is necessary to promote an intercultural approach that allows dialogue between traditional knowledge and formal medicine, respecting the cultural identity of communities and strengthening practices that contribute to the well-being of both mother and newborn.

Emergent Questions

1. Interviewer: How did you learn to be a midwife?
Interviewee (Ms. Sara): I learned from my mother. She was a midwife, and from a young age I accompanied her when she attended women. By observing and helping, I gradually learned, because in my community there was no health center.

2. Interviewer: Why was midwifery important in your village?
Interviewee: It was very important because there was no health post and the hospital was far away. There were no vehicles; we only traveled by donkey or horse. To avoid risking the lives of the mother and the baby, it was necessary for someone to know how to assist births.

3. Interviewer: What care did you provide to the baby after birth?
Interviewee: We cut the cord with clean glass, wrapped it in cotton, and cared for it so it would not get infected. Water should not enter the navel. We also wrapped the baby with a *chumpi* so it would grow straight.

4. Interviewer: What rituals were performed to protect the newborn?
Interviewee: We painted the tip of the nose red so the baby would have a good gaze. When we went to a wake, we placed rue or molle leaves in the baby's armpit so that "qayqa" would not affect them. That was to protect them from bad energies.

5. Interviewer: What care should the mother follow after childbirth?
Interviewee: She should not carry weight or fetch water for at least three weeks. She needed to rest in bed. To produce milk, we gave her sheep or cow broth and fennel tea.

6. Interviewer: Why did you stop practicing midwifery?
Interviewee: When I moved to the city of Ayacucho, I stopped practicing. Also, when I accepted the Christian faith, I understood that some rituals no longer aligned with my beliefs, so I stopped practicing them.
